# Supplementary material for: The comparative biogeography of Philippine geckos challenges predictions from a paradigm of climate‐driven vicariant diversification across an island archipelago
Source: Evolution. 2019 May 9;73(6):1151–67. doi: 10.1111/evo.13754 (PMC6767427; doi:10.1111/evo.13754)
Supplement: Supplementary file 1 — Table S1. The data for all samples included in the three RADseq libraries are included in a separate tab‐delimited text file available from the Dryad Digital Repository (https://datadryad.org/resource/doi:10.5061/dryad.n34d4m7/3). Table S2. The data for all samples included in the 16 pairs of populations analyzed in this study are included in a separate tab‐delimited text file available from the Dryad Digital Repository (https://datadryad.org/resource/doi:10.5061/dryad.n34d4m7/4). Table S3. Settings used for assembling loci for each pair of gekkonid populations. Table S4. Per‐site nucleotide diversity within (π1 and π2) and between (πbetween) pairs of Cyrtodactylus and Gekko populations, calculated from the RADseq data using the SeqSift Python package (https://github.com/joaks1/SeqSift), which relies on Biopython (https://biopython.org/). Figure S1. Bathymetry contours around the Philippine Islands at varying depths. Figure S2. Animation of approximate sea‐level changes in the Philippine Islands over the last 430,000 years. Figure S3. Approximate prior (light bars) and posterior (dark bars) probabilities of numbers of divergence events across pairs of Cyrtodactylus populations under three different priors on the concentration parameter of the Dirichlet process. Figure S4. Approximate marginal posterior densities of divergence times for each pair of Cyrtodactylus populations under three different priors on the concentration parameter of the Dirichlet process. Figure S5. Approximate prior (light bars) and posterior (dark bars) probabilities of numbers of divergence events across pairs of Cyrtodactylus populations under four different combinations of prior on divergence times (rows) and recoding or removing polyallelic characters (columns). Figure S6. Approximate marginal posterior densities of divergence times for each pair of Cyrtodactylus populations under four different combinations of prior on divergence times (rows) and recoding or removing polyallelic characters [file EVO-73-1151-s001.pdf]

Supporting Information

Title: The comparative biogeography of Philippine geckos challenges predictions from a paradigm of climate-driven vicariant diversification across an island archipelago

Authors: Jamie R. Oaks Corresponding author: joaks@auburn.edu[1], Cameron D. Siler[2], and Rafe M. Brown[3]

[1]Department of Biological Sciences & Museum of Natural History, Auburn University, Auburn, Alabama 36849, USA
[2]Sam Noble Oklahoma Museum of Natural History and Department of Biology, University of Oklahoma, Norman, Oklahoma 73072-7029
[3]Biodiversity Institute and Department of Ecology and Evolutionary Biology, University of Kansas, Lawrence, Kansas 66045, USA

Table S1. The data for all samples included in the three RADseq libraries are included in a separate tab-delimited text file available from the Dryad Digital Repository (https://datadryad.org/resource/doi:10.5061/dryad.n34d4m7/3).

Table S2. The data for all samples included in the 16 pairs of populations analyzed in this study are included in a separate tab-delimited text file available from the Dryad Digital Repository (https://datadryad.org/resource/doi:10.5061/dryad.n34d4m7/4). This is a subset of the data in Table S1.

Table S3. Settings used for assembling loci for each pair of gekkonid populations.

**ipyrad setting**	**Value**
assembly_method	denovo
datatype	rad
restriction_overhang	TATG,
max_low_qual_bases	4
phred_Qscore_offset	33
mindepth_statistical	6
mindepth_majrule	6
maxdepth	10000
clust_threshold	0.85
max_barcode_mismatch	0
filter_adapters	1
filter_min_trim_len	35
max_alleles_consens	2
max_Ns_consens	4
max_Hs_consens	5
min_samples_locus	4
max_SNPs_locus	20
max_Indels_locus	8
max_shared_Hs_locus	0.5
trim_reads	0, 0, 0, 0
trim_loci	0, 0, 0, 0

Table S4. Per-site nucleotide diversity within (π_1 and π_2) and between ($\pi_{between}$) pairs of *Cyrtodactylus* and *Gekko* populations, calculated from the RADseq data using the SeqSift Python package (https://github.com/joaks1/SeqSift), which relies on Biopython (https://biopython.org/). The within-population nucleotide diversity (π_1 and π_2) is followed by the number of individuals sampled from the population in parentheses.

Species	Island 1	Island 2	π_1	π_2	$\pi_{between}$
C. annulatus	Bohol	Camiguin Sur	0.00118 (4)	0.00136 (4)	0.00303
C. baluensis-redimiculus	Palawan	Kinabalu	0.00135 (4)	0.00432 (3)	0.02475
C. gubaot-sumuroi	Samar	Leyte	0.00573 (5)	0.00223 (5)	0.00653
C. philippinicus	Luzon	Babuyan Claro	0.00131 (2)	0.00028 (2)	0.00226
C. philippinicus	Luzon	Camiguin Norte	0.00102 (3)	0.00068 (4)	0.00349
C. philippinicus	Polillo	Luzon	0.00226 (5)	0.00211 (5)	0.00429
C. philippinicus	Panay	Negros	0.00177 (3)	0.00095 (2)	0.00335
C. philippinicus	Sibuyan	Tablas	0.00136 (3)	0.00209 (3)	0.00319
G. crombota-rossi	Babuyan Claro	Calayan	0.00101 (5)	0.00111 (5)	0.00340
G. gigante	South Gigante	North Gigante	0.00167 (3)	0.00127 (4)	0.00149
G. mindorensis	Lubang	Luzon	0.00183 (5)	0.00119 (4)	0.00851
G. mindorensis	Panay	Masbate	0.00137 (4)	0.00130 (3)	0.00407
G. mindorensis	Negros	Panay	0.00103 (3)	0.00177 (5)	0.00259
G. porosus	Sabtang	Batan	0.00092 (4)	0.00115 (4)	0.00172
G. romblon	Romblon	Tablas	0.00178 (5)	0.00218 (2)	0.00349
G. sp. a-sp. b	Camiguin Norte	Dalupiri	0.00096 (5)	0.00111 (5)	0.00262

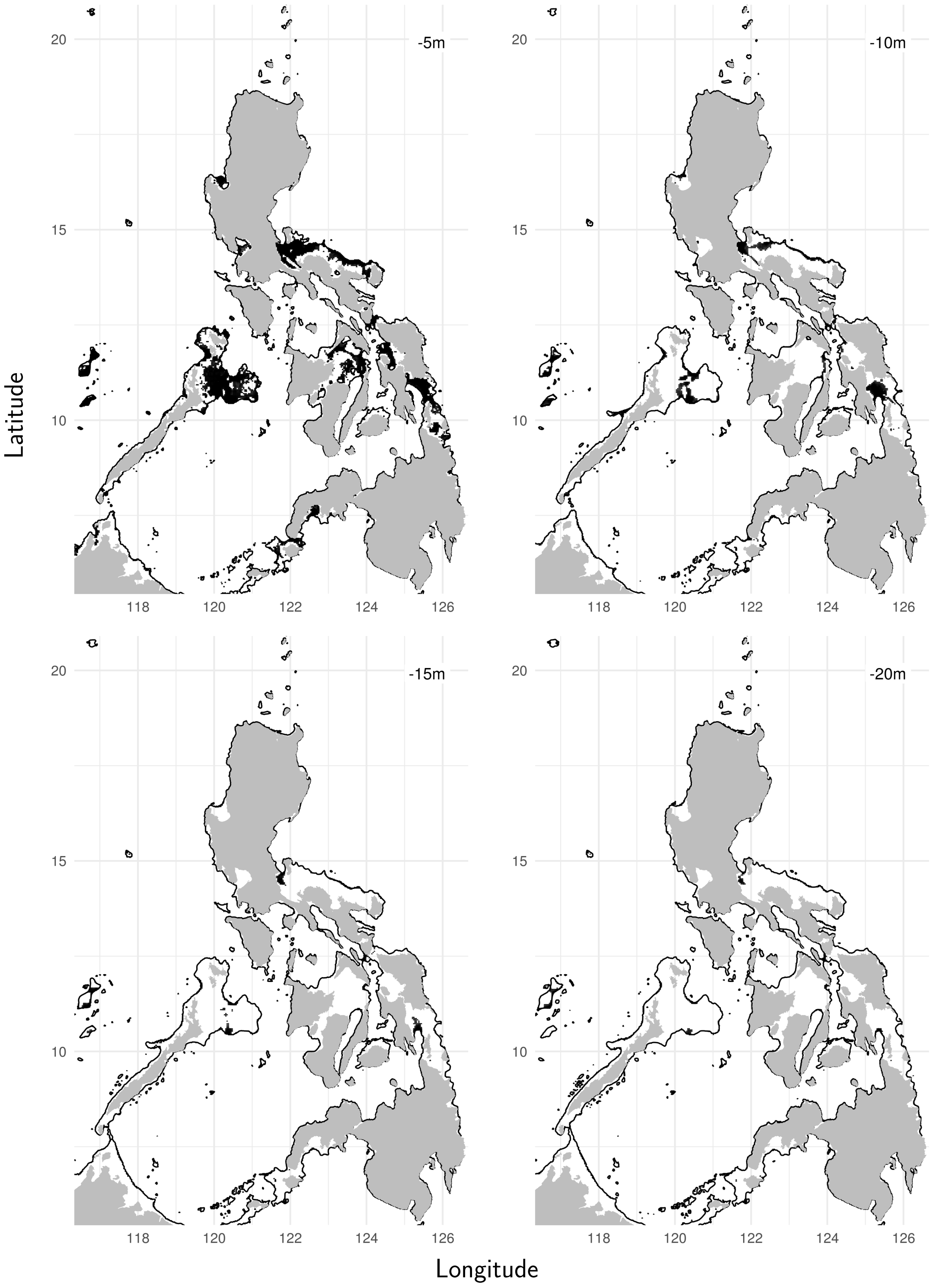

Figure S1. Bathymetry contours around the Philippine Islands at varying depths. Black lines show the contour associated with the depth indicated in the upper right of each plot. Contours are based on data from the ETOPO1 1-arc-minute global relief model (Amante and Eakins, 2009). Figure generated with marmap version 1.0.2 (Pante and Simon-Bouhet, 2013) and ggplot2 Version 2.2.1 (Wickham, 2009).

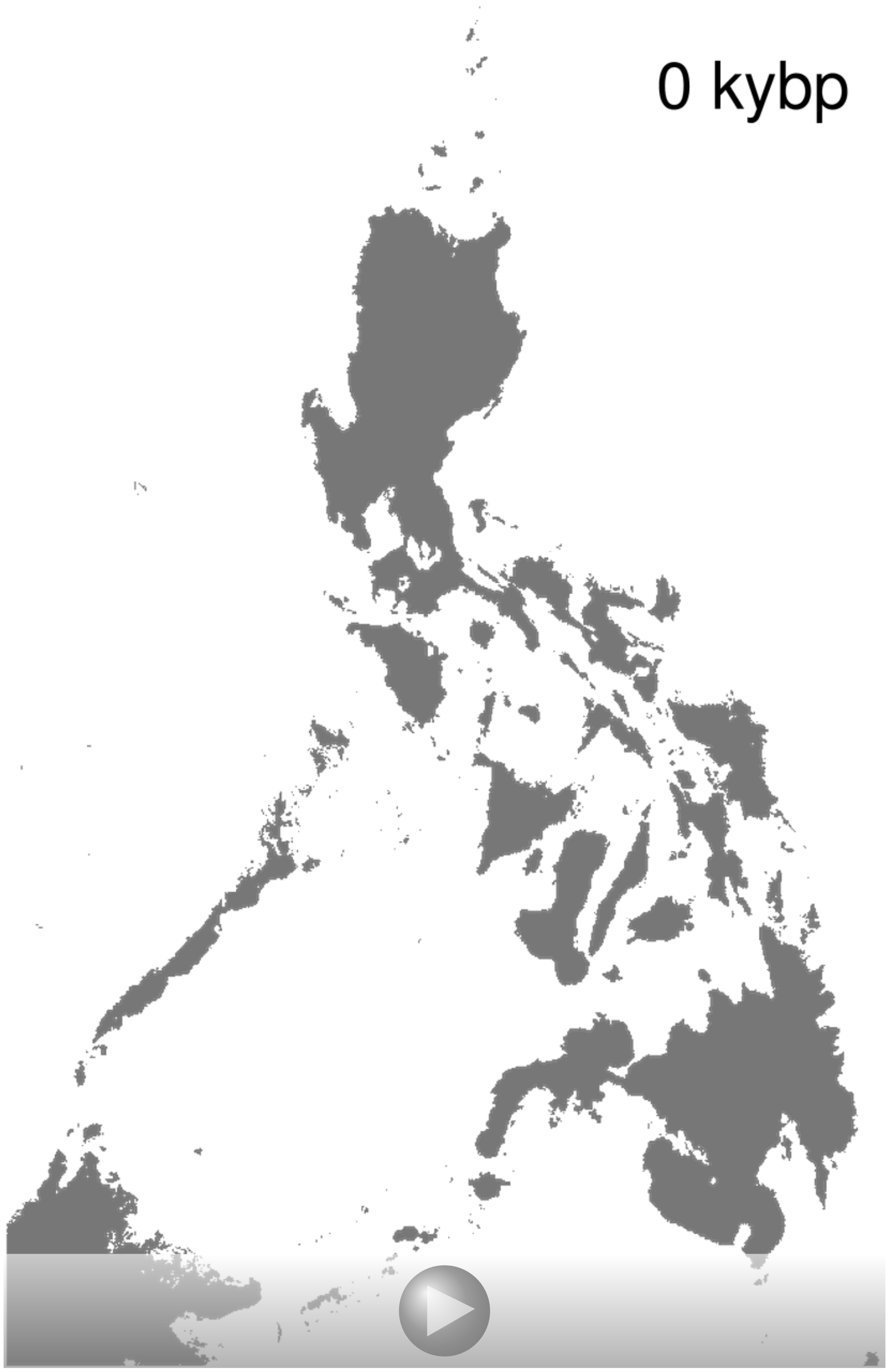

Figure S2. Animation of approximate sea-level changes in the Philippine Islands over the last 430,000 years. Sea-level estimates are from the projection of Spratt and Lisiecki (2016) based on seven reconstructions. Bathymetry data are from the ETOPO1 1-arc-minute global relief model (Amante and Eakins, 2009). Animation generated using marmap version 1.0.2 (Pante and Simon-Bouhet, 2013), ggplot2 Version 2.2.1 (Wickham, 2009), ImageMagick Version 6.9.10-8 Q16 x86_64 20180723, and FFmpeg Version 4.0.2-2. The source code for generating the plot is available at `https://github.com/phyletica/animating-sea-level-change`. This animation can also be viewed at `https://youtu.be/NjGdCezUvw8` or downloaded from the Dryad Digital Repository: `https://doi.org/10.5061/dryad.n34d4m7/5`.

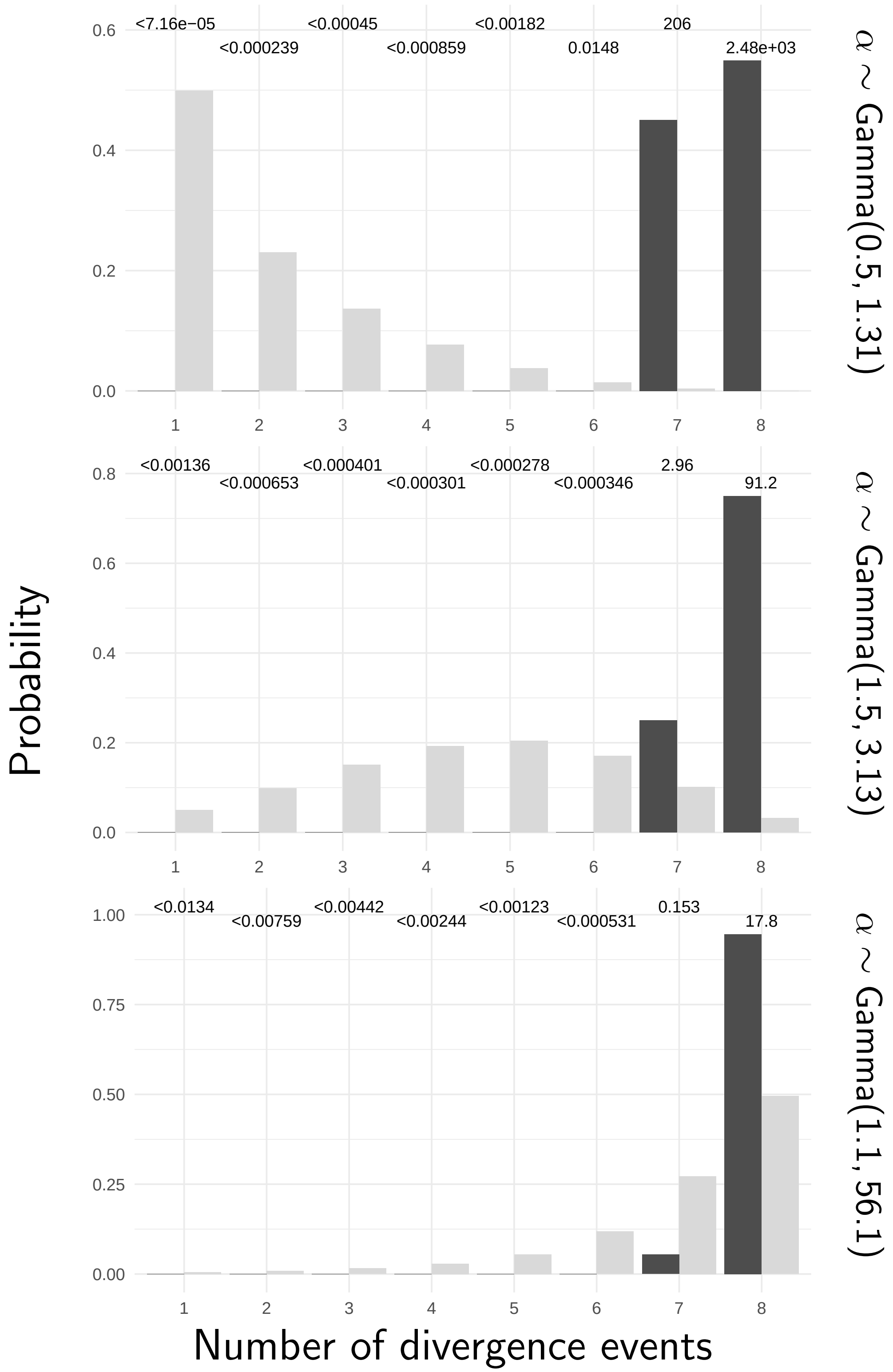

Figure S3. Approximate prior (light bars) and posterior (dark bars) probabilities of numbers of divergence events across pairs of *Cyrtodactylus* populations under three different priors on the concentration parameter of the Dirichlet process. Bayes factors for each number of divergence times is given above the corresponding bars. Each Bayes factor compares the corresponding number of events to all other possible numbers of divergence events. Figure generated with ggplot2 Version 2.2.1 (Wickham, 2009).

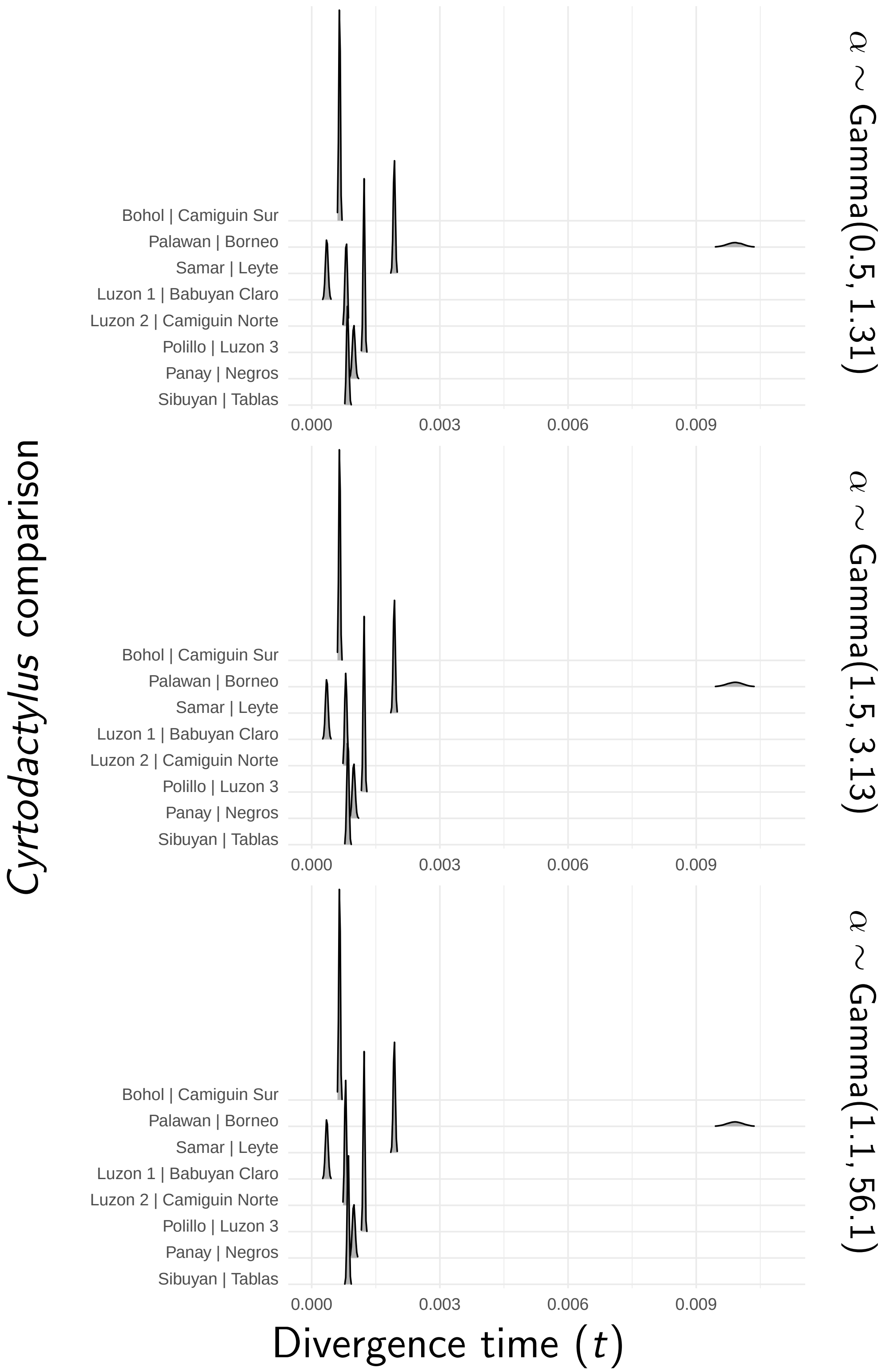

Figure S4. Approximate marginal posterior densities of divergence times for each pair of *Cyrtodactylus* populations under three different priors on the concentration parameter of the Dirichlet process. Figure generated with ggridges Version 0.4.1 (Wilke, 2018) and ggplot2 Version 2.2.1 (Wickham, 2009).

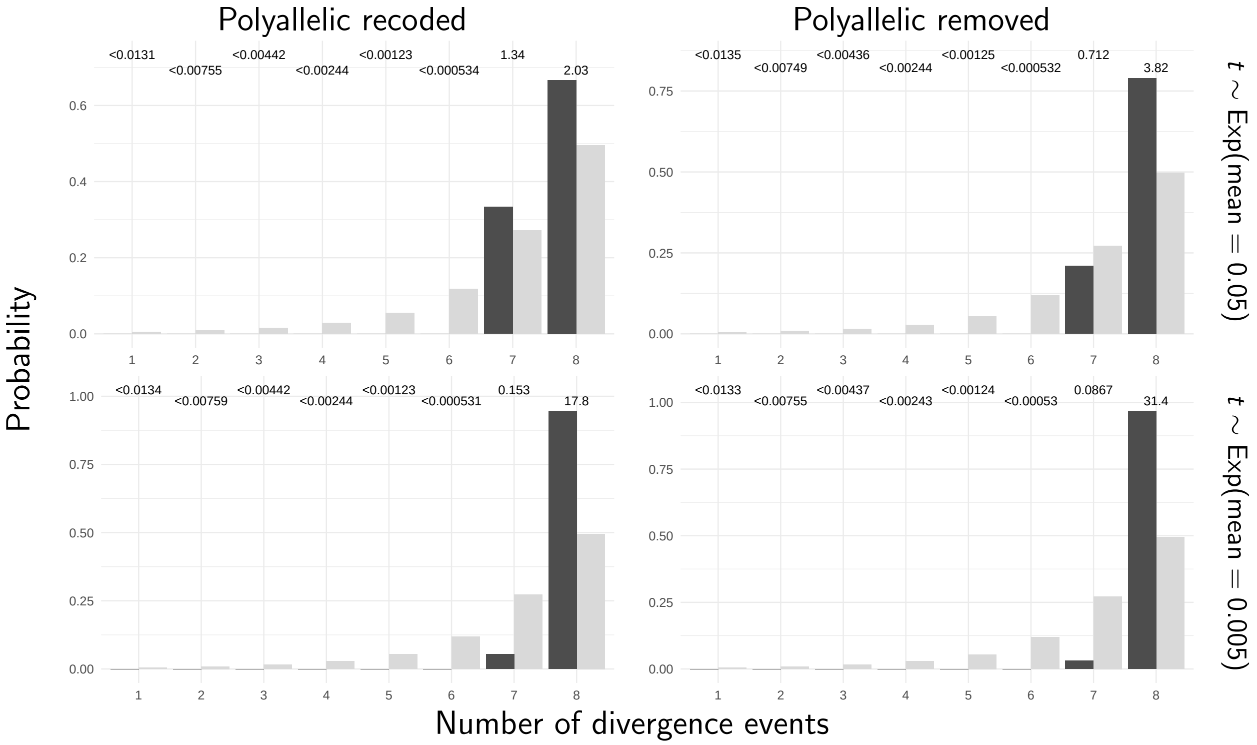

Figure S5. Approximate prior (light bars) and posterior (dark bars) probabilities of numbers of divergence events across pairs of *Cyrtodactylus* populations under four different combinations of prior on divergence times (rows) and recoding or removing polyallelic characters (columns). Bayes factors for each number of divergence times is given above the corresponding bars. Each Bayes factor compares the corresponding number of events to all other possible numbers of divergence events. Figure generated with ggplot2 Version 2.2.1 (Wickham, 2009).

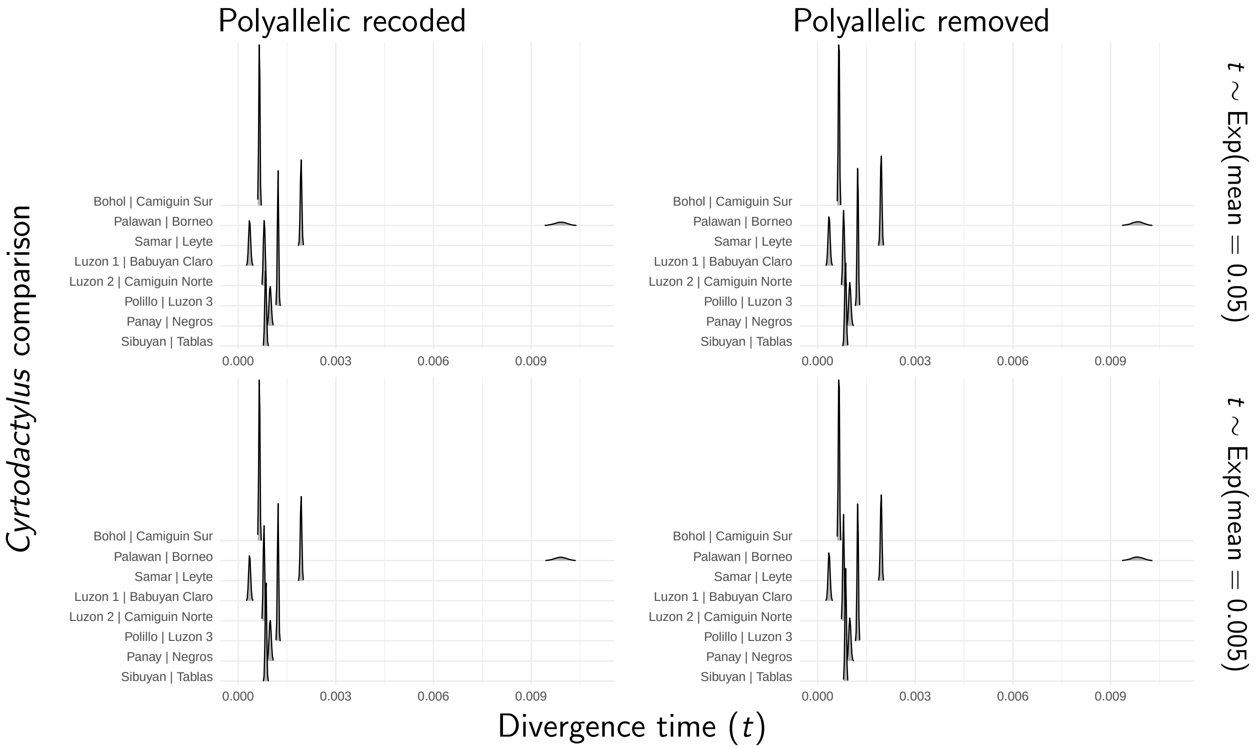

Figure S6. Approximate marginal posterior densities of divergence times for each pair of *Cyrtodactylus* populations under four different combinations of prior on divergence times (rows) and recoding or removing polyallelic characters (columns). Figure generated with ggridges Version 0.4.1 (Wilke, 2018) and ggplot2 Version 2.2.1 (Wickham, 2009).

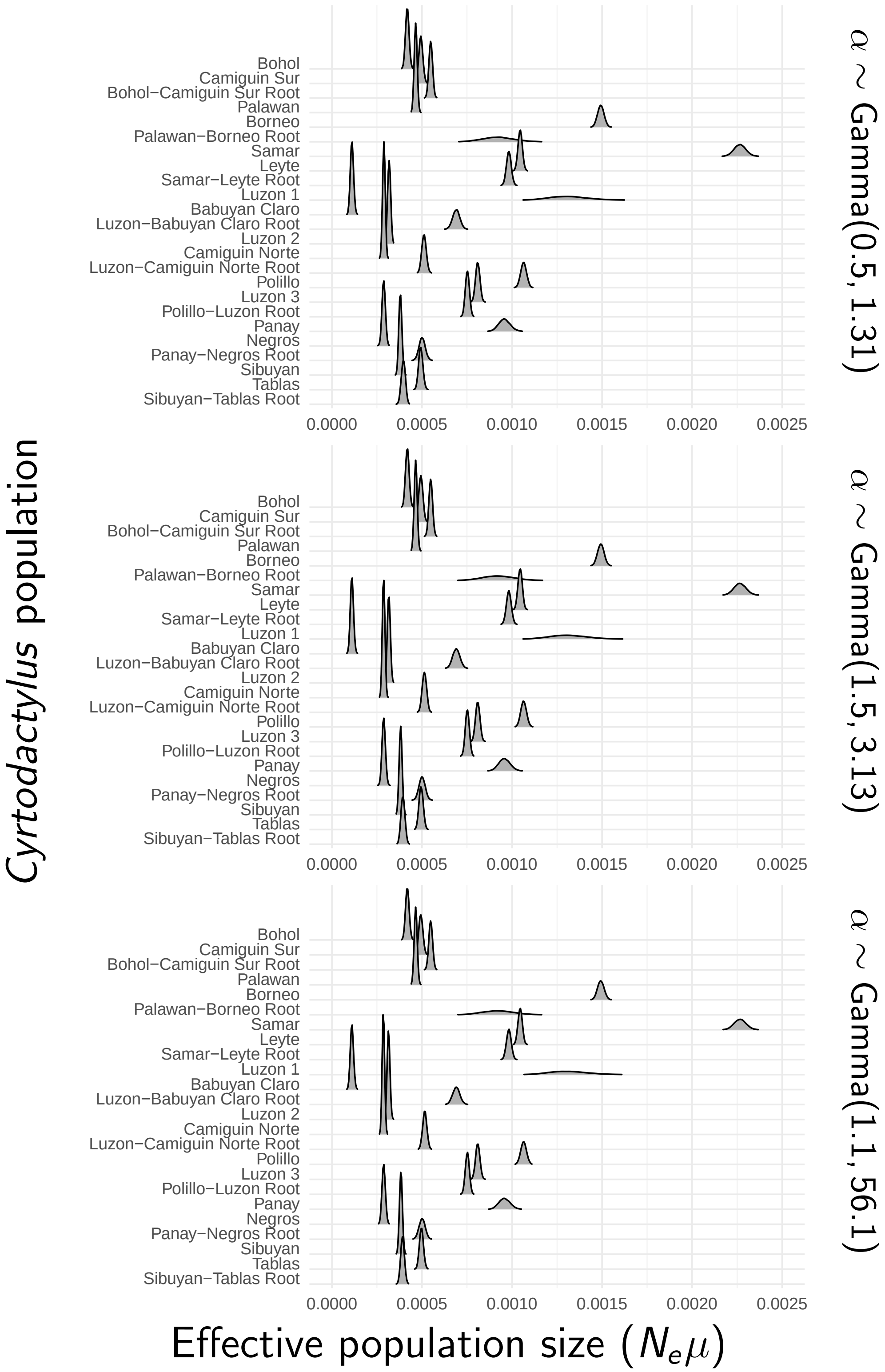

Figure S7. Approximate marginal posterior densities of population sizes for each pair of *Cyrtodactylus* populations under three different priors on the concentration parameter of the Dirichlet process. Figure generated with ggridges Version 0.4.1 (Wilke, 2018) and ggplot2 Version 2.2.1 (Wickham, 2009).

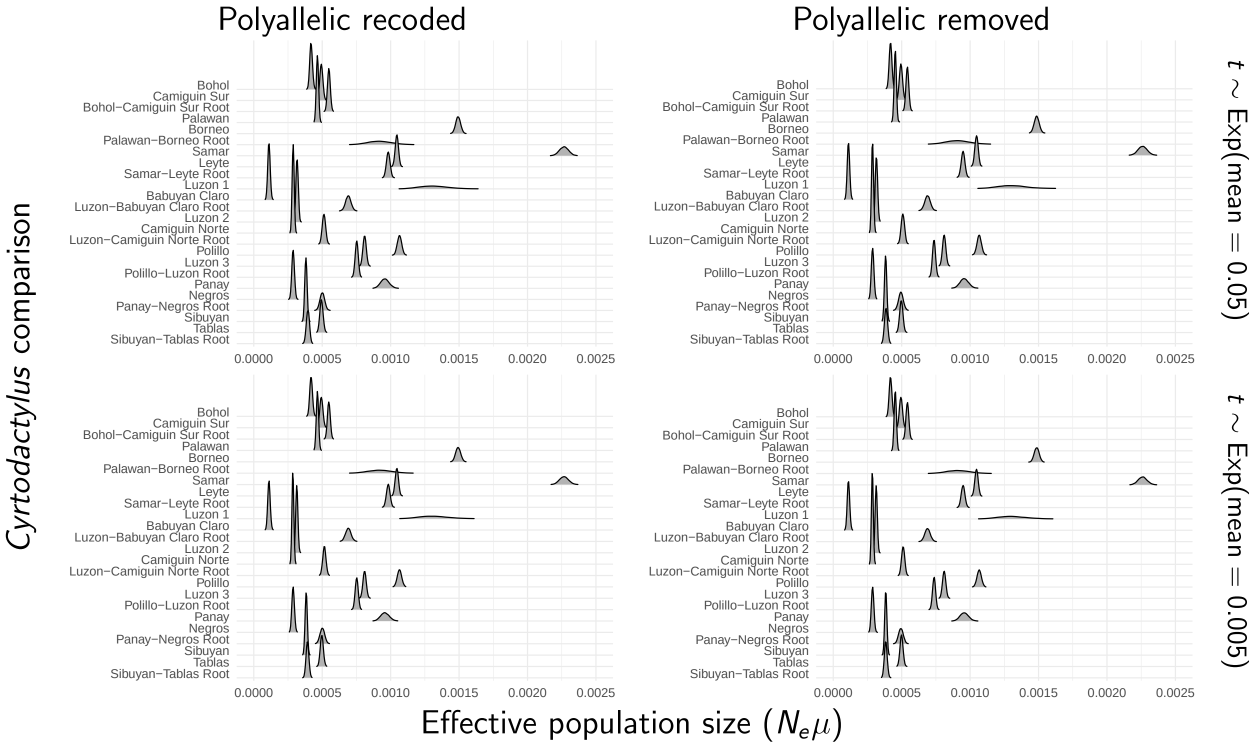

Figure S8. Approximate marginal posterior densities of population sizes for each pair of *Cyrtodactylus* populations under four different combinations of prior on divergence times (rows) and recoding or removing polyallelic characters (columns). Figure generated with ggridges Version 0.4.1 (Wilke, 2018) and ggplot2 Version 2.2.1 (Wickham, 2009).

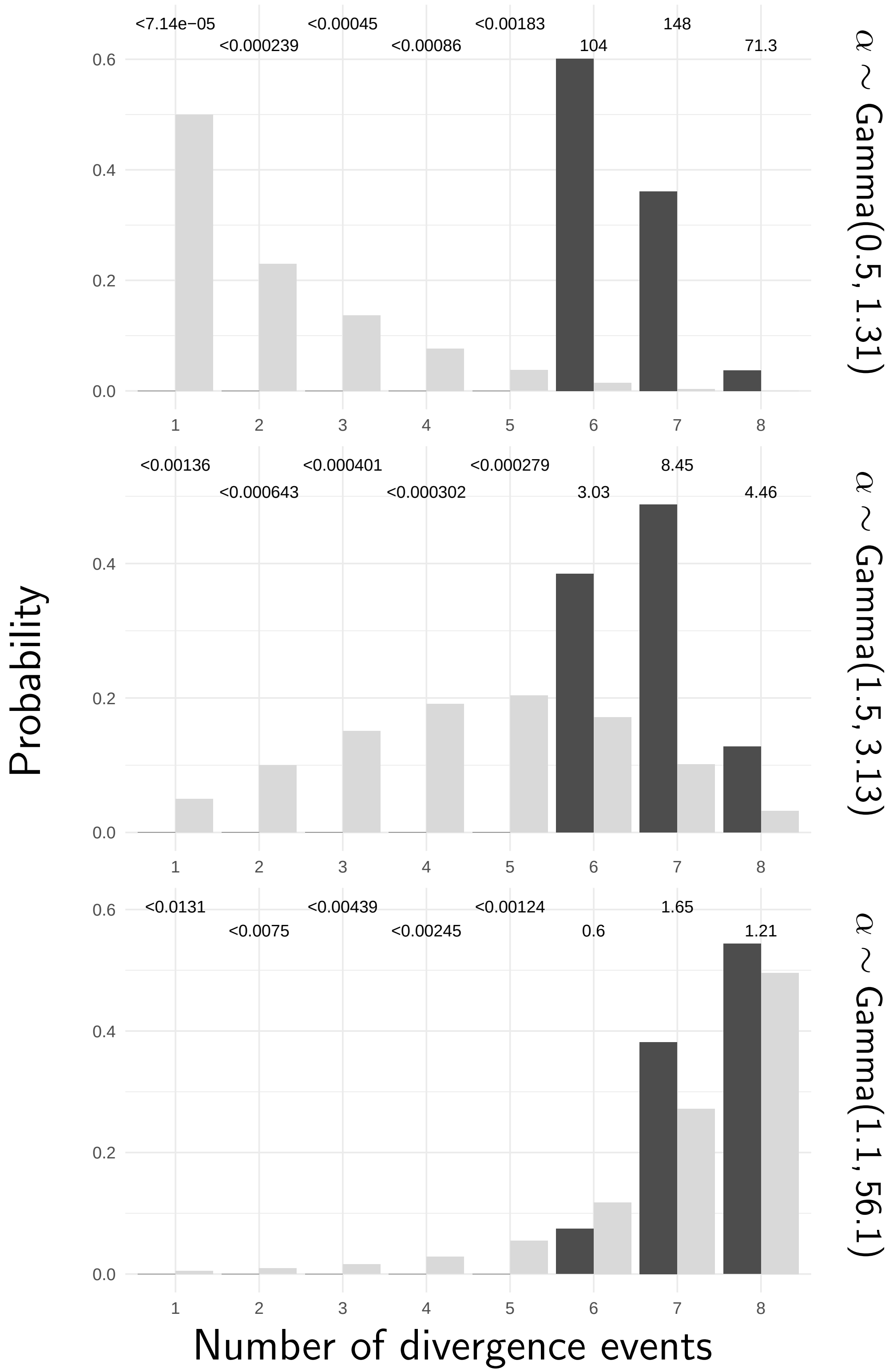

Figure S9. Approximate prior (light bars) and posterior (dark bars) probabilities of numbers of divergence events across pairs of *Gekko* populations under three different priors on the concentration parameter of the Dirichlet process. Bayes factors for each number of divergence times is given above the corresponding bars. Each Bayes factor compares the corresponding number of events to all other possible numbers of divergence events. Figure generated with ggplot2 Version 2.2.1 (Wickham, 2009).

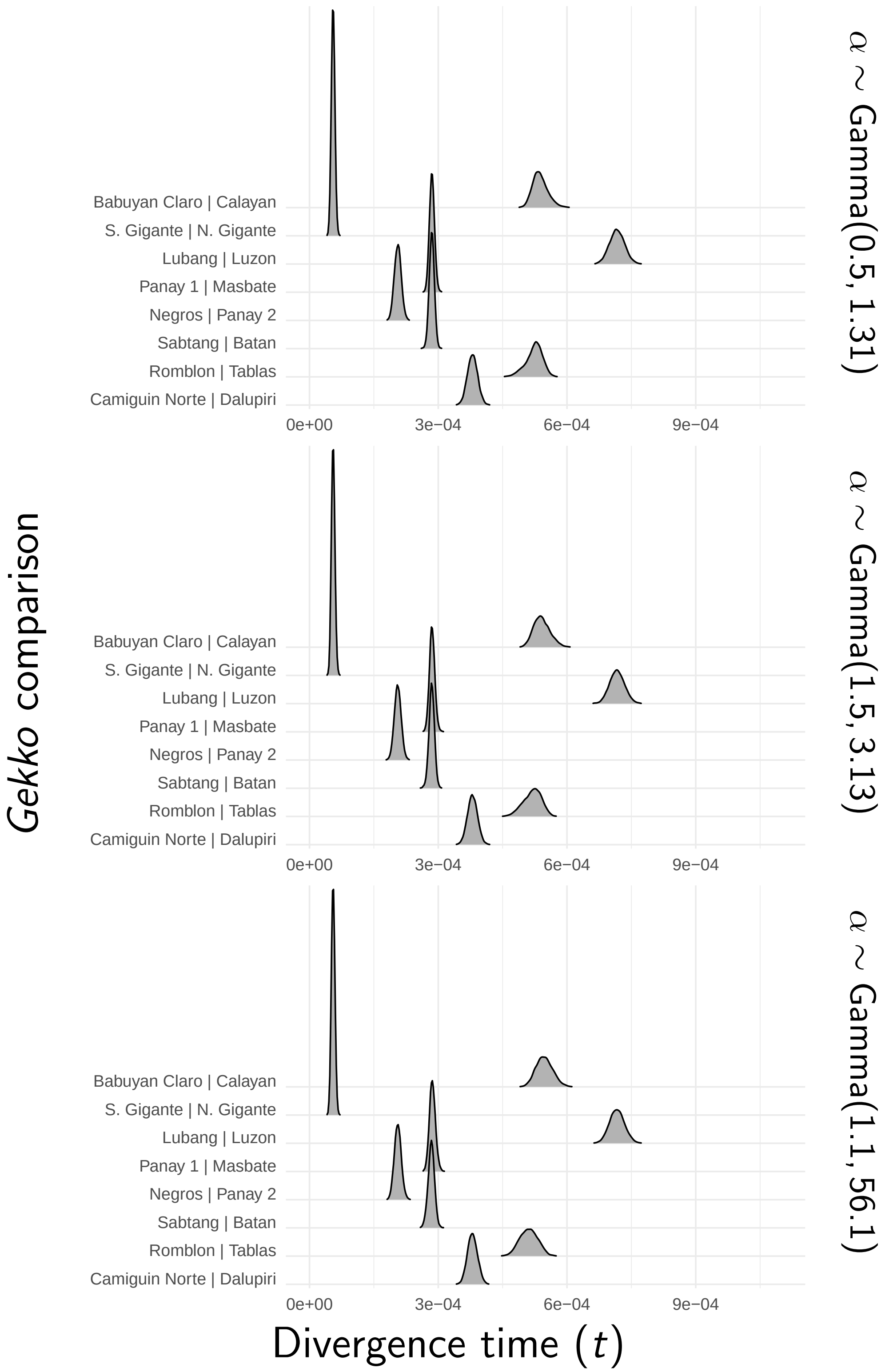

Figure S10. Approximate marginal posterior densities of divergence times for each pair of *Gekko* populations under three different priors on the concentration parameter of the Dirichlet process. Figure generated with ggridges Version 0.4.1 (Wilke, 2018) and ggplot2 Version 2.2.1 (Wickham, 2009).

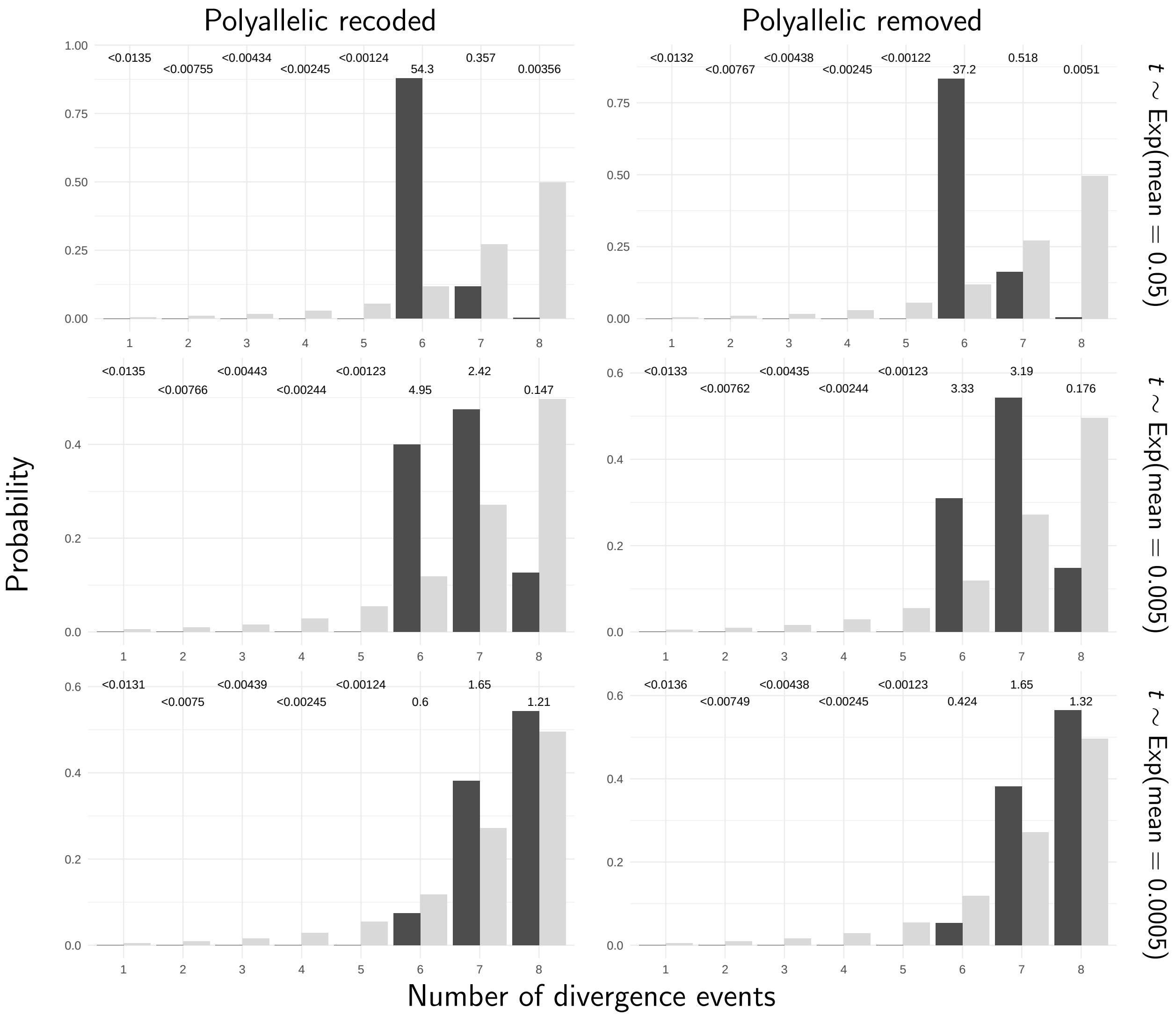

Figure S11. Approximate prior (light bars) and posterior (dark bars) probabilities of numbers of divergence events across pairs of *Gekko* populations under six different combinations of prior on divergence times (rows) and recoding or removing polyallelic characters (columns). Bayes factors for each number of divergence times is given above the corresponding bars. Each Bayes factor compares the corresponding number of events to all other possible numbers of divergence events. Figure generated with ggplot2 Version 2.2.1 (Wickham, 2009).

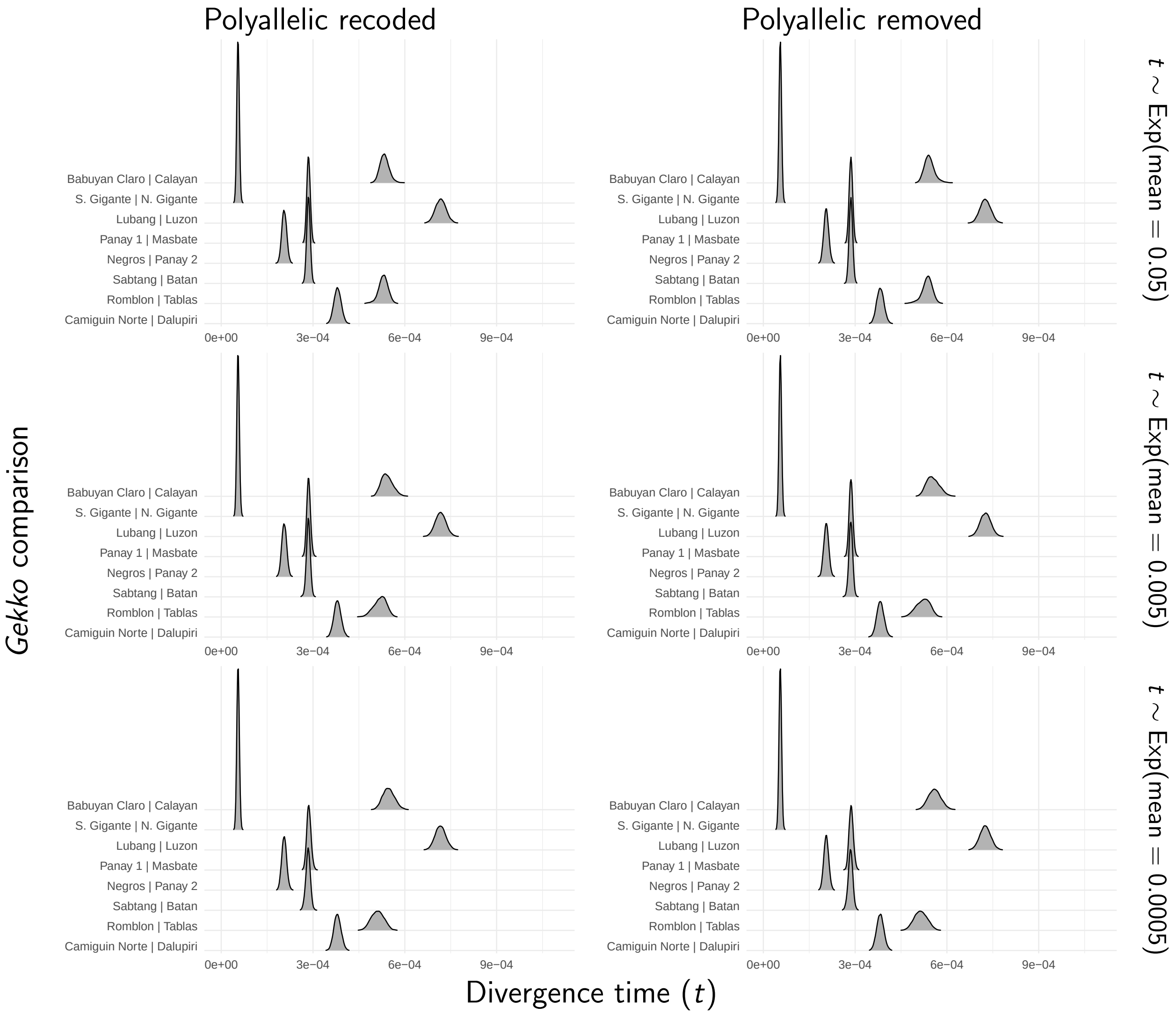

Figure S12. Approximate marginal posterior densities of divergence times for each pair of *Gekko* populations under six different combinations of prior on divergence times (rows) and recoding or removing polyallelic characters (columns). Figure generated with ggridges Version 0.4.1 (Wilke, 2018) and ggplot2 Version 2.2.1 (Wickham, 2009).

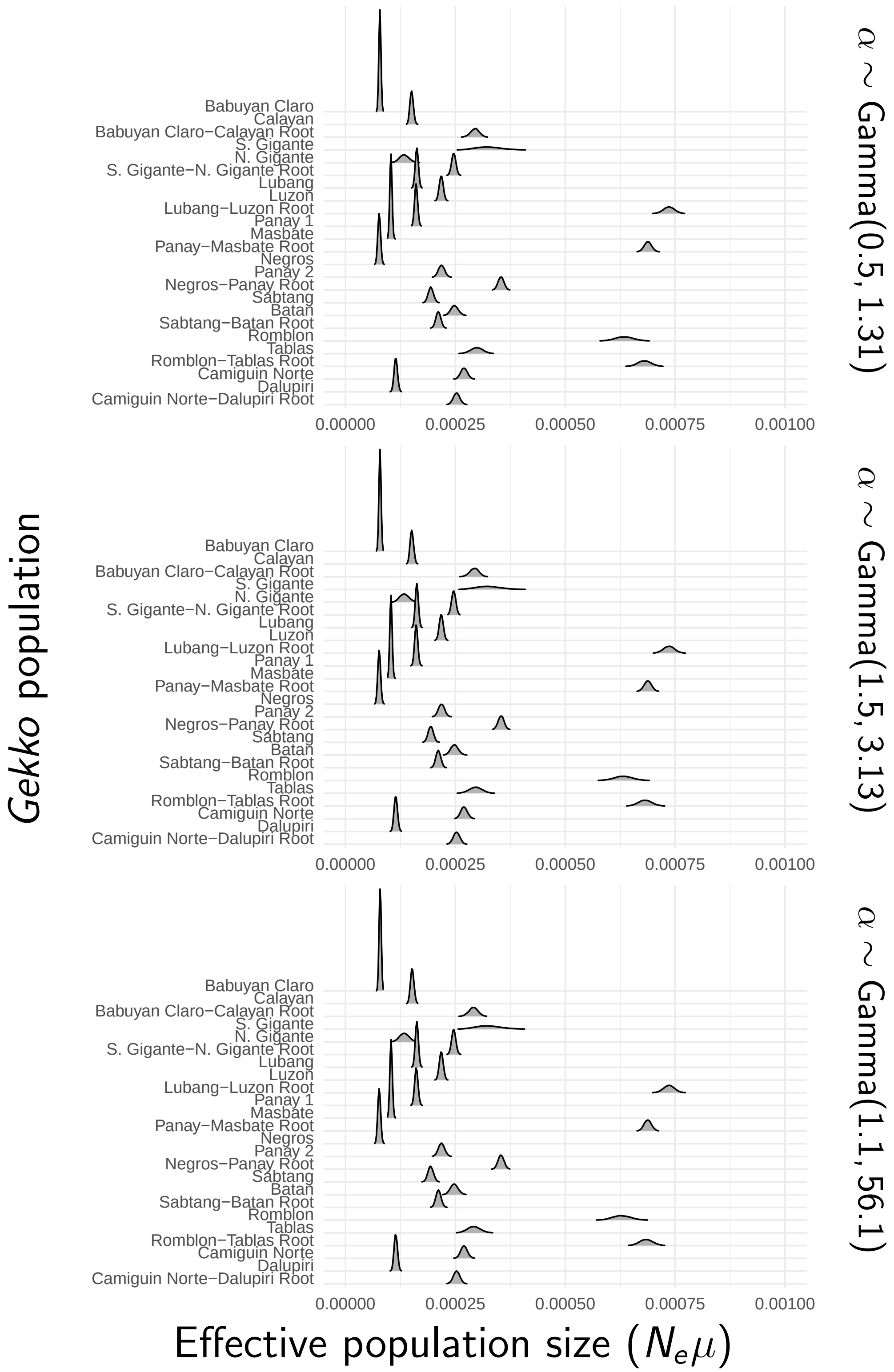

Figure S13. Approximate marginal posterior densities of population sizes for each pair of *Gekko* populations under three different priors on the concentration parameter of the Dirichlet process. Figure generated with ggridges Version 0.4.1 (Wilke, 2018) and ggplot2 Version 2.2.1 (Wickham, 2009).

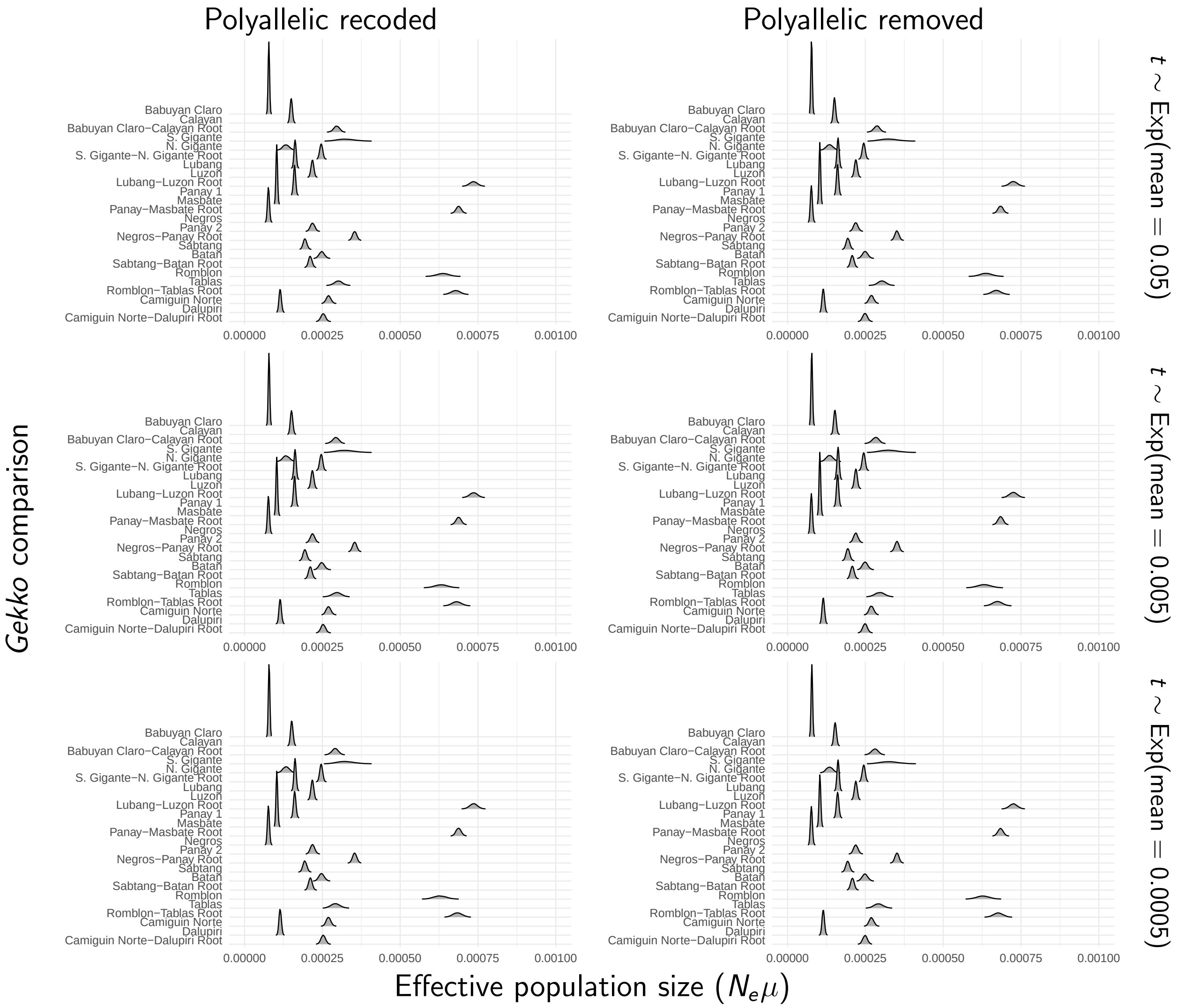

Figure S14. Approximate marginal posterior densities of population sizes for each pair of *Gekko* populations under six different combinations of prior on divergence times (rows) and recoding or removing polyallelic characters (columns). Figure generated with ggridges Version 0.4.1 (Wilke, 2018) and ggplot2 Version 2.2.1 (Wickham, 2009).

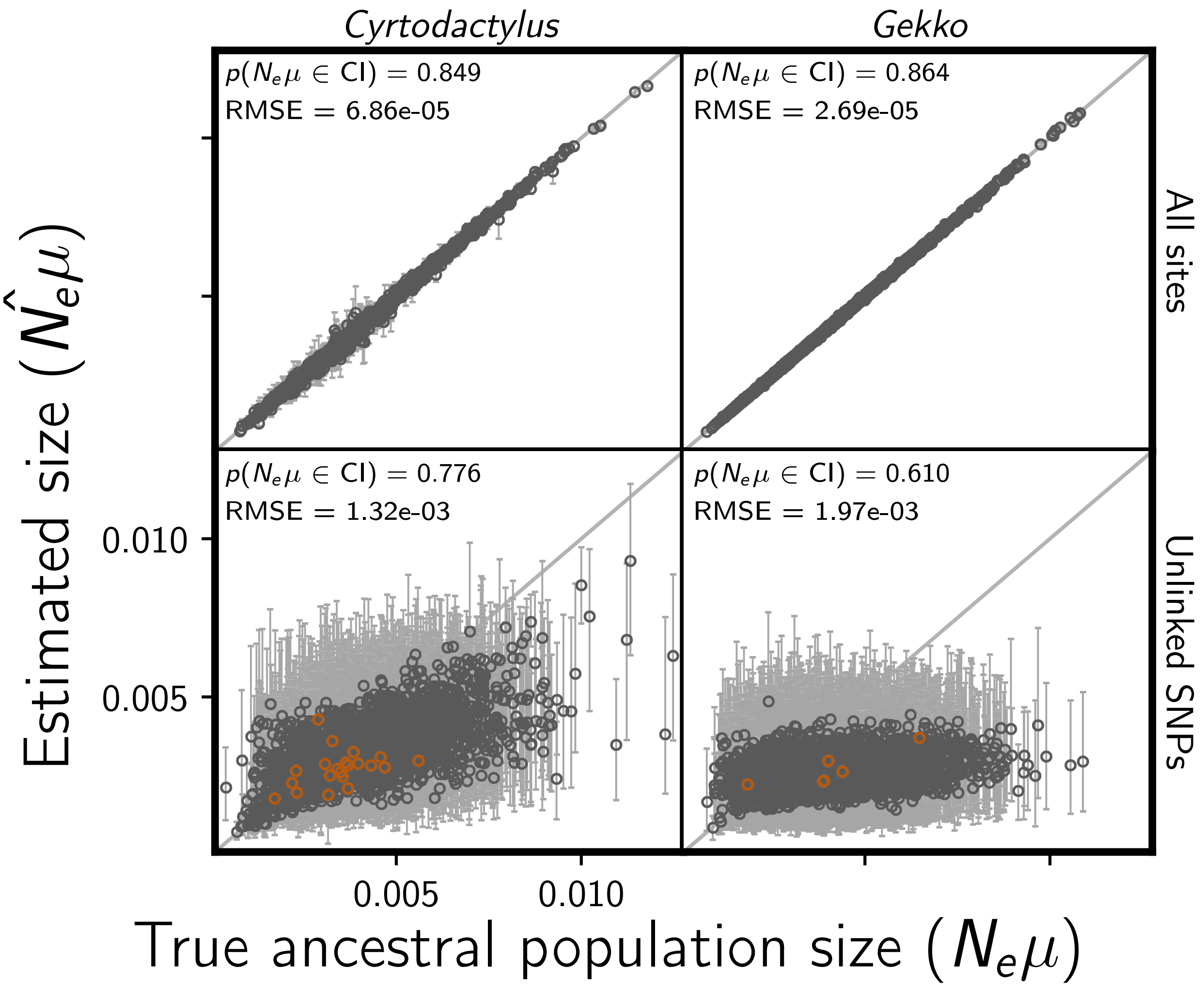

Figure S15. The accuracy and precision of `ecoevolity` estimates of the ancestral population size (scaled by the mutation rate) when applied to data simulated to match our *Cyrtodactylus* (left) and *Gekko* (right) RADseq data sets with all sites (top) or only one SNP per locus (bottom). Each circle and associated error bars represent the posterior mean and 95% credible interval. Estimates for which the potential-scale reduction factor was greater than 1.2 (Brooks and Gelman, 1998) are highlighted in orange. Each plot consists of 4000 estimates—500 simulated data sets, each with eight pairs of populations. For each plot, the root-mean-square error (RMSE) and the proportion of estimates for which the 95% credible interval contained the true value—$p(N_e\mu \in \mathrm{CI})$—is given. Figure generated with matplotlib Version 2.0.0 (Hunter, 2007).

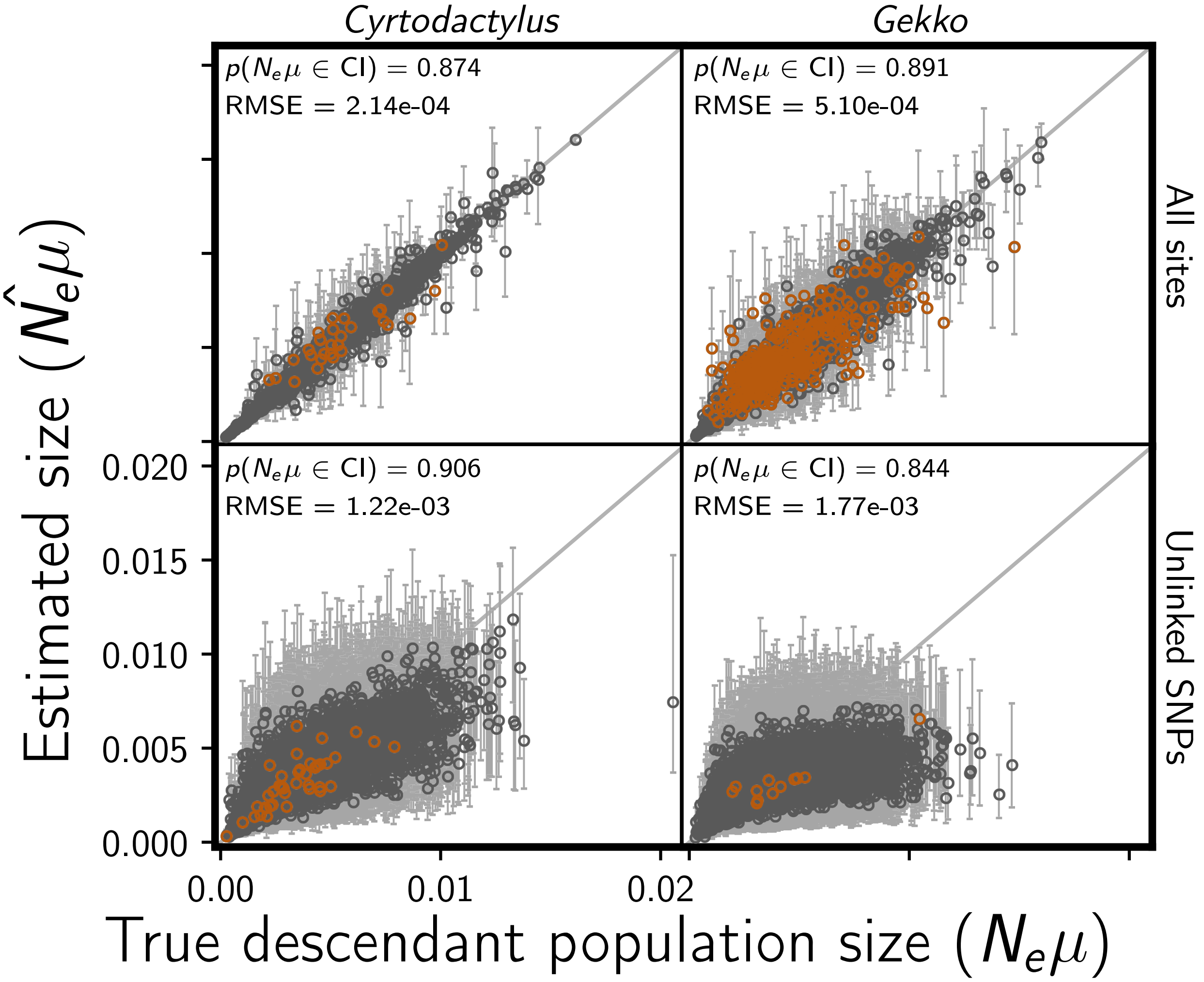

Figure S16. Accuracy and precision of `ecoevolity` estimates of the descendant population sizes (scaled by the mutation rate) when applied to data simulated to match empirical *Cyrtodactylus* (left) and *Gekko* (right) RADseq data sets with all sites (top) or only one SNP per locus (bottom). Each circle and associated error bars represents the posterior mean and 95% credible interval. Estimates for which the potential-scale reduction factor was greater than 1.2 (Brooks and Gelman, 1998) are highlighted in orange. Each plot consists of 8000 estimates—500 simulated data sets, each with eight pairs of populations. For each plot, the root-mean-square error (RMSE) and the proportion of estimates for which the 95% credible interval contained the true value—$p(N_e\mu \in \mathrm{CI})$—is given. Figure generated with matplotlib Version 2.0.0 (Hunter, 2007).

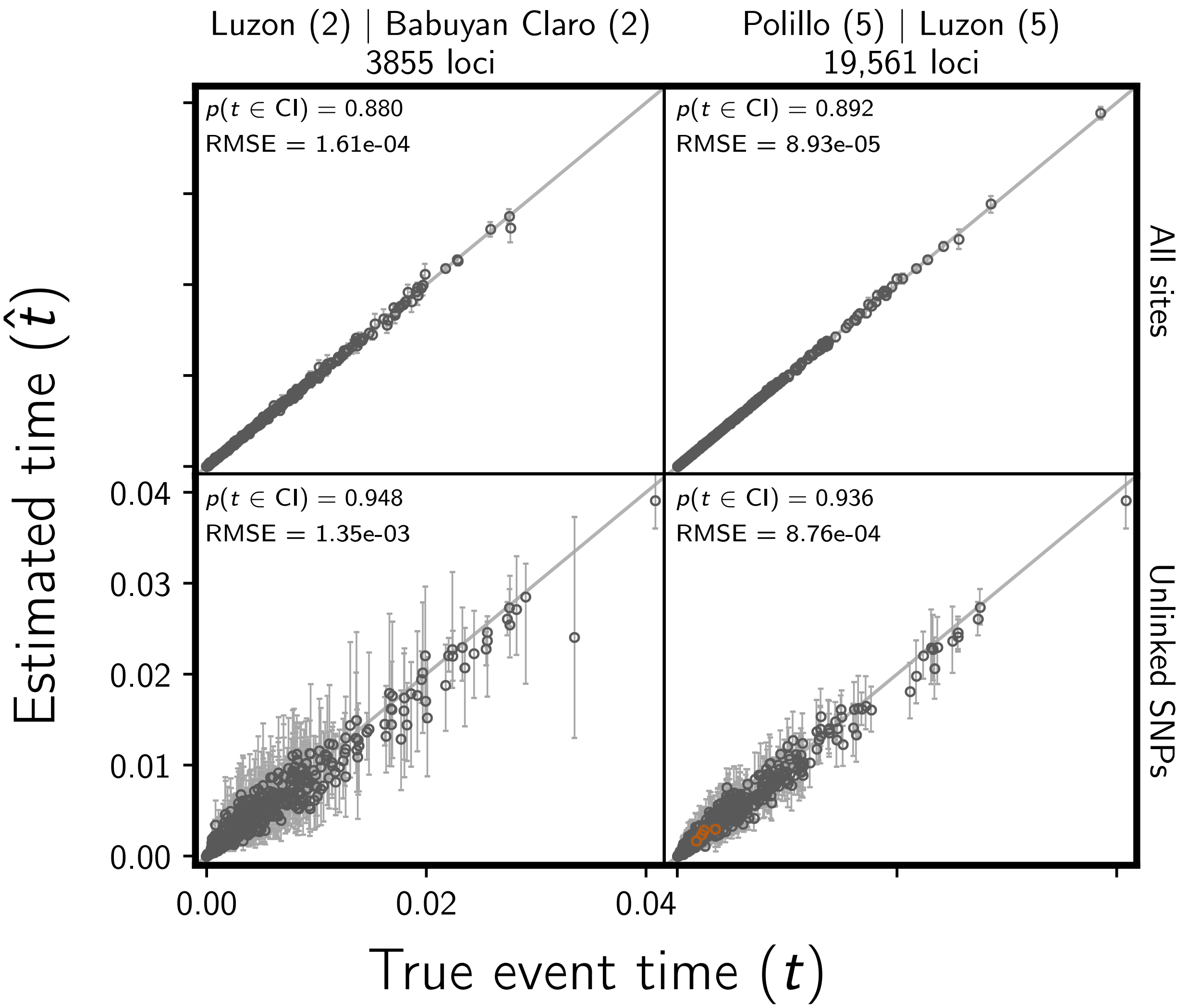

Figure S17. Accuracy and precision of `ecoevolity` divergence-time estimates (in units of expected subsitutions per site) when applied to data simulated to match empirical RADseq data sets sampled from the pairs of *Cyrtodactylus philippinicus* populations from the islands of (left) Luzon and Babuyan Claro and (right) Polillo and Luzon (a subset of the results plotted in Figure 6). Results are shown for `ecoevolity` analyses of data sets that contain all sites (top), or only one SNP per locus (bottom). The number of individuals sampled from each island population is indicated in parentheses at the top of each column of plots. Results for these two pairs of populations are plotted separately here to compare divergence-times estimated from data sets with large differences in the number of sampled individuals and loci. Each circle and associated error bars represents the posterior mean and 95% credible interval for the time that a pair of populations diverged. Estimates for which the potential-scale reduction factor was greater than 1.2 (Brooks and Gelman, 1998) are highlighted in orange. Each plot consists of 500 estimates—one from each of the 500 simulated data sets. For each plot, the root-mean-square error (RMSE) and the proportion of estimates for which the 95% credible interval contained the true value—$p(t \in \mathrm{CI})$—is given. Figure generated with matplotlib Version 2.0.0 (Hunter, 2007).
