# Supplementary material for: The comparative biogeography of Philippine geckos challenges predictions from a paradigm of climate‐driven vicariant diversification across an island archipelago
Source: Evolution. 2019 May 9;73(6):1151–67. doi: 10.1111/evo.13754 (PMC6767427; doi:10.1111/evo.13754)
Supplement: Supplementary file 2 — Journal Club Slides [file EVO-73-1151-s002.pdf]

The comparative biogeography of Philippine geckos challenges predictions from a paradigm of climate-driven vicariant diversification across an island archipelago

Jamie R. Oaks[1] Cameron D. Siler[2] Rafe M. Brown[3]

[1]Department of Biological Sciences & Museum of Natural History, Auburn University, Auburn, Alabama 36849, USA

[2]Sam Noble Oklahoma Museum of Natural History and Department of Biology, University of Oklahoma, Norman, Oklahoma 73072-7029

[3]Biodiversity Institute and Department of Ecology and Evolutionary Biology, University of Kansas, Lawrence, Kansas 66045, USA

May 3, 2019

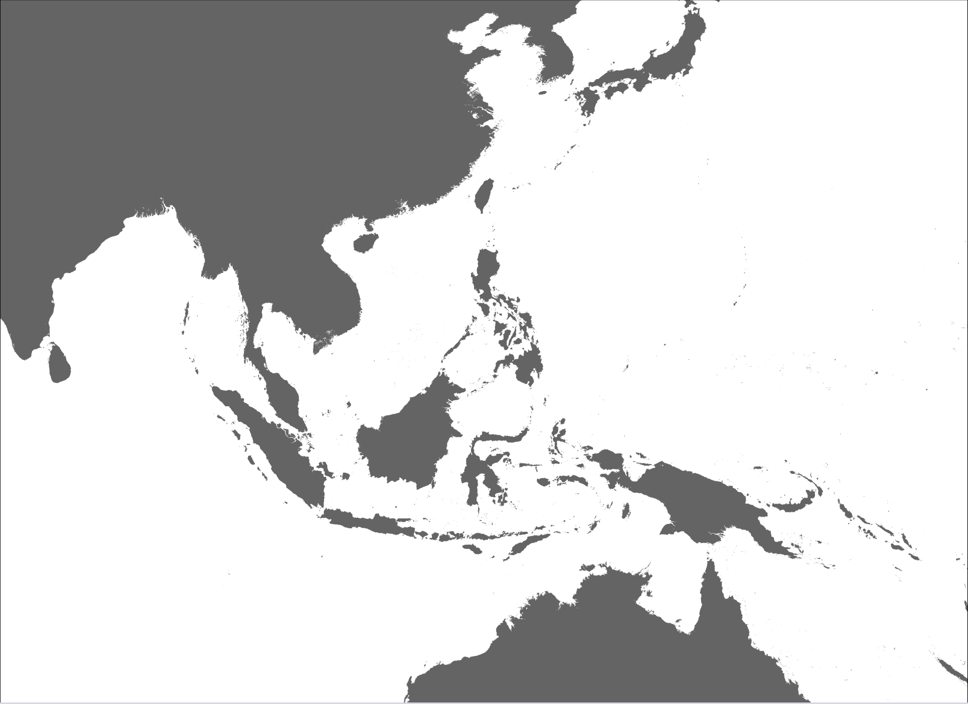

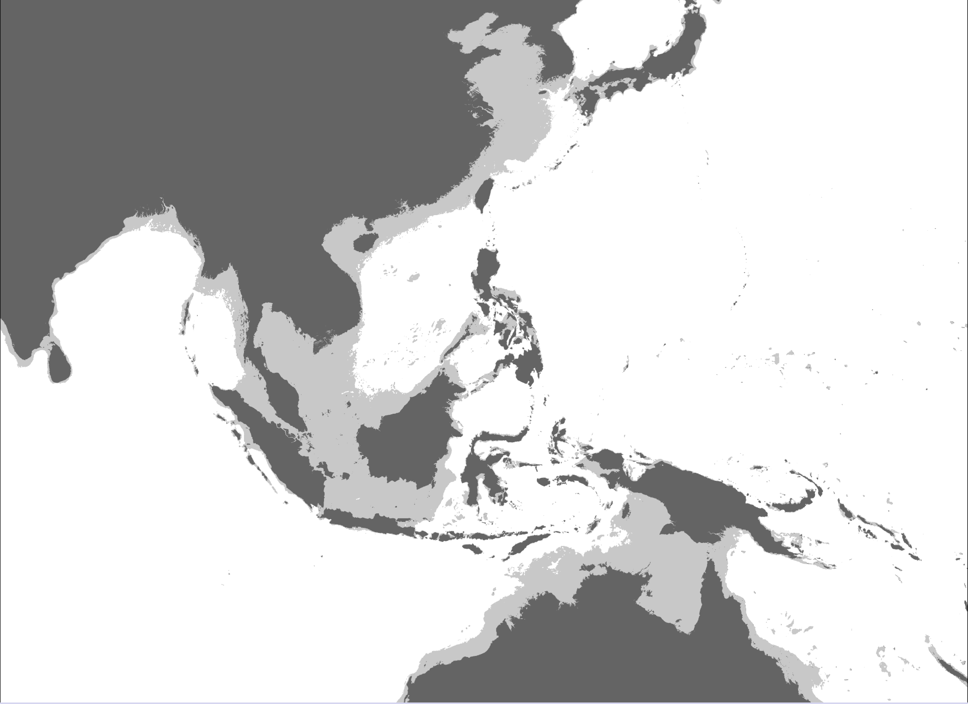

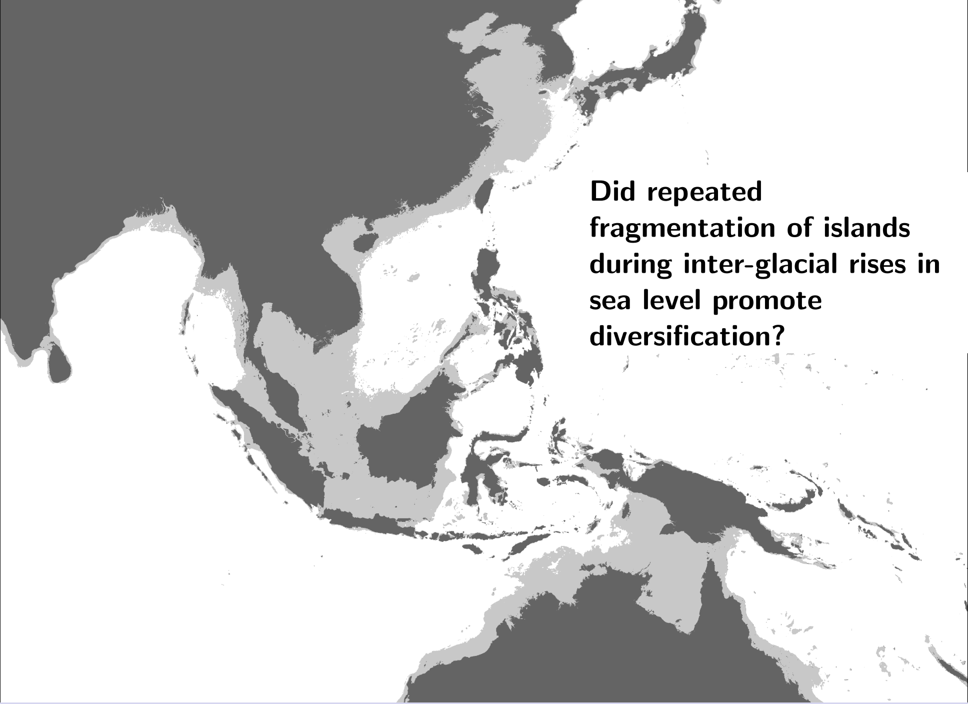

Did repeated fragmentation of islands during inter-glacial rises in sea level promote diversification?

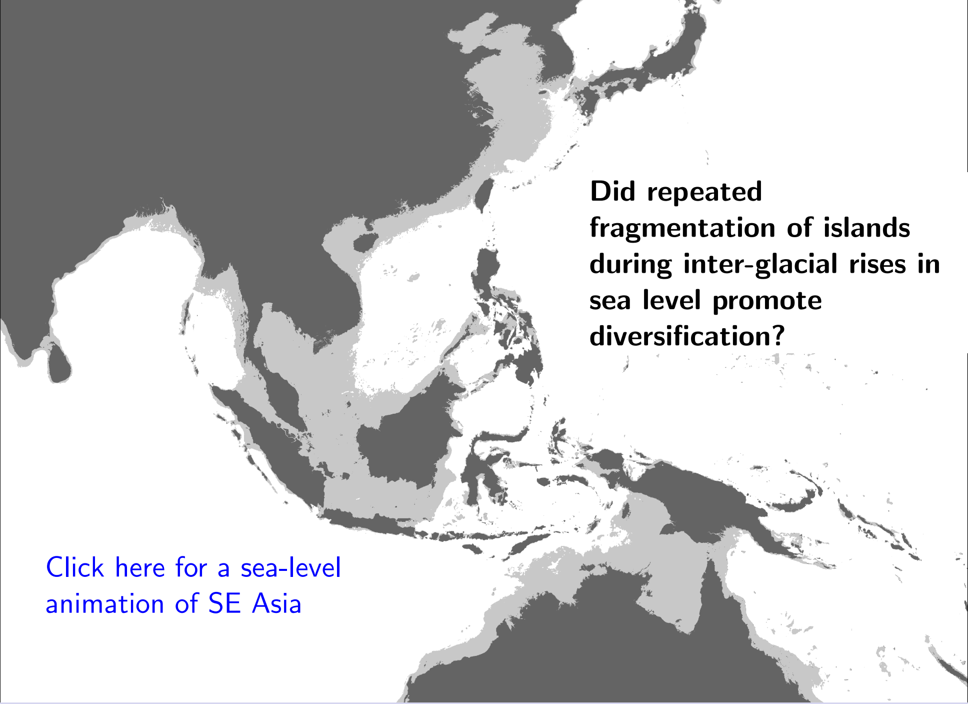

Did repeated fragmentation of islands during inter-glacial rises in sea level promote diversification?

Click here for a sea-level animation of SE Asia

"Species-pump" Hypothesis Repeated climate-driven fragmentation of the Philippine Islands was a primary mechanism of speciation for terrestrial fauna

"Species-pump" Hypothesis Repeated climate-driven fragmentation of the Philippine Islands was a primary mechanism of speciation for terrestrial fauna

Prediction Taxa co-distributed across islands within the same Pleistocene aggregate island complex (PAIC) will have divergence times that tend to be clustered around times when sea levels fragmented the islands

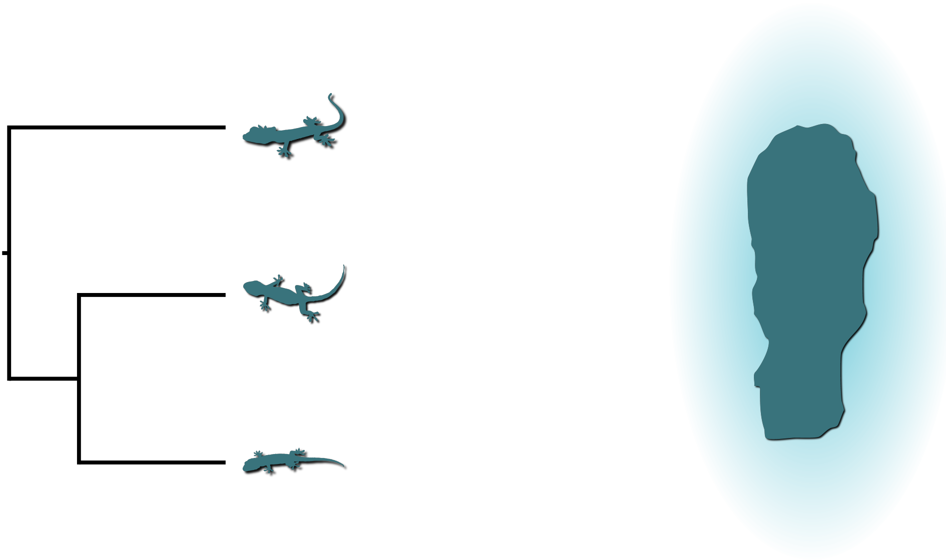

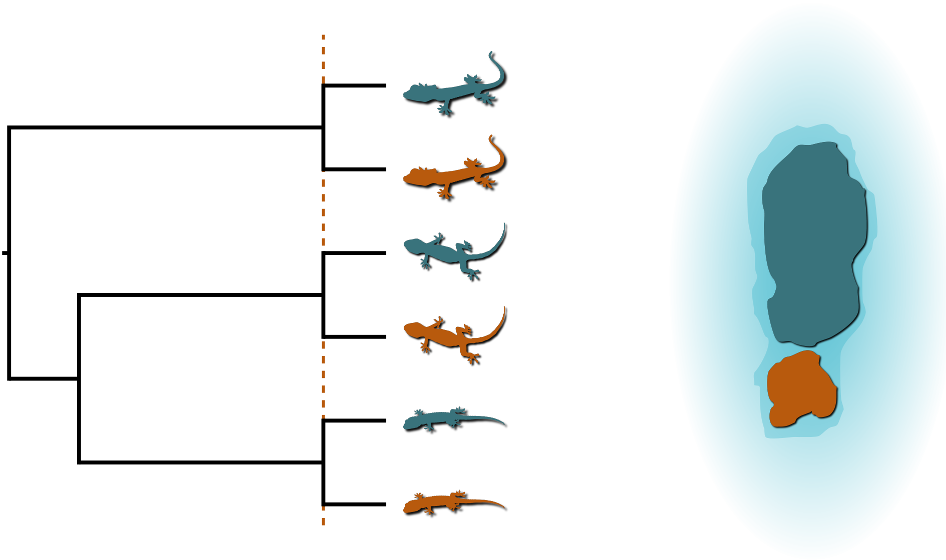

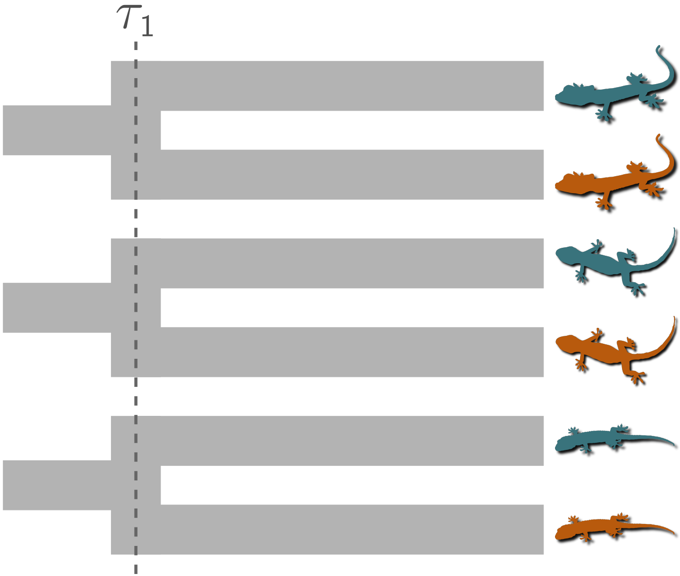
τ_1

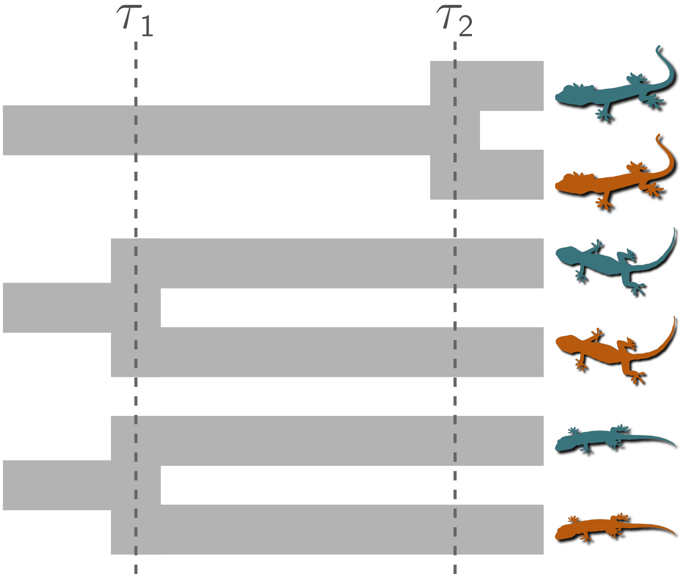

τ_1
τ_2

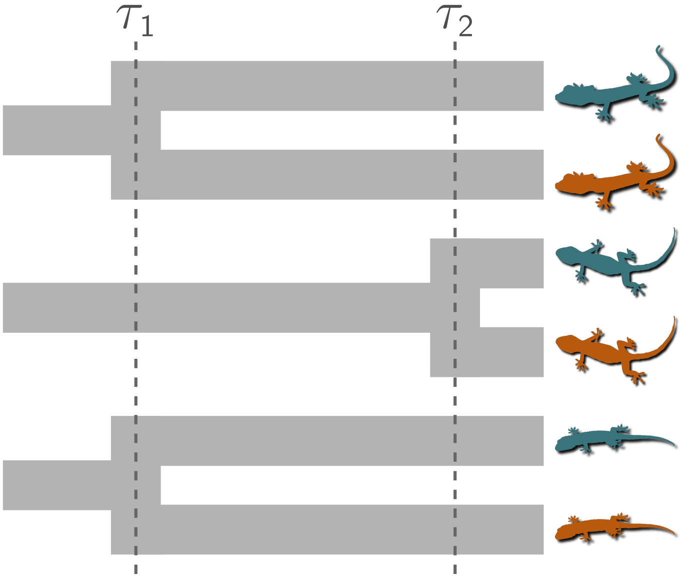

τ_1
τ_2

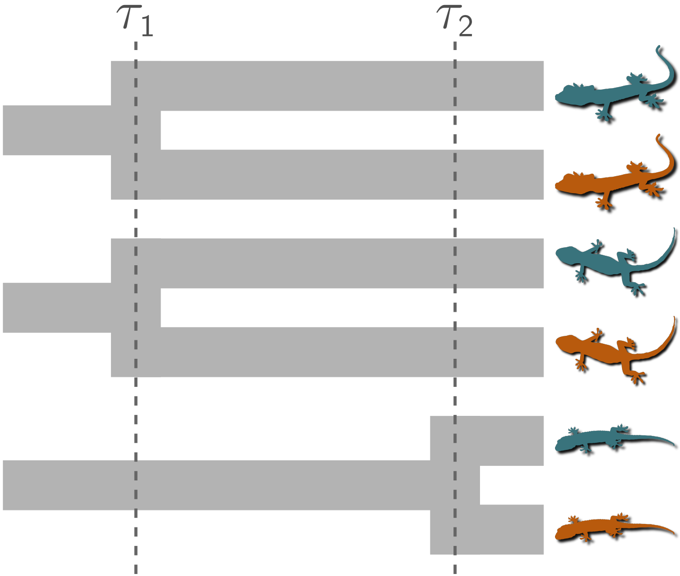
τ_1
τ_2

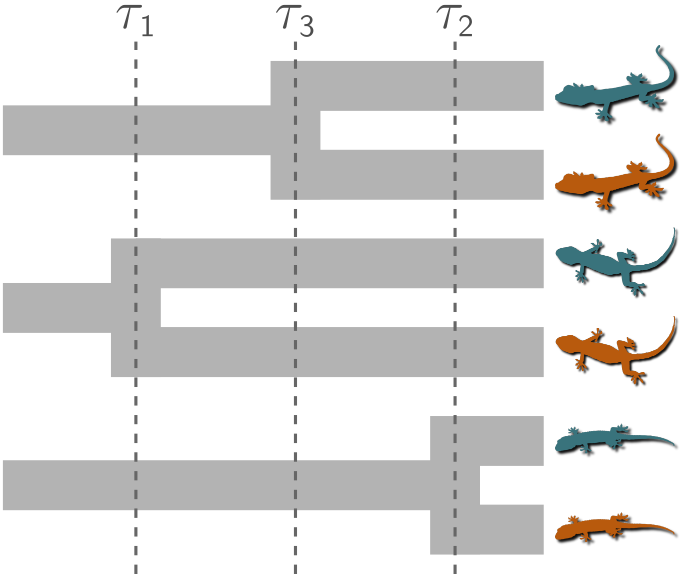

τ_1
τ_3
τ_2

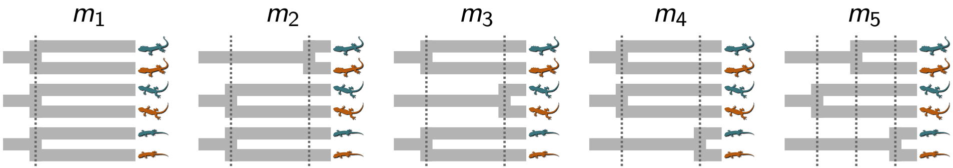

m_1
m_2
m_3
m_4
m_5

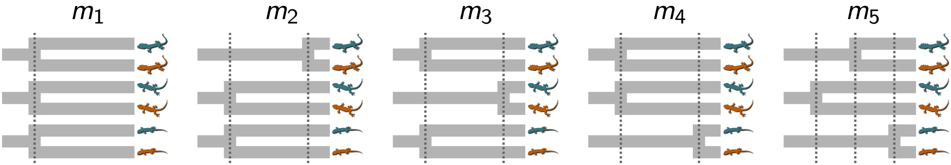

We want to infer the model and divergence times given genetic data

Previous tests of "species-pump"

- Oaks et al. (2013)[1] collected mitochondrial DNA sequences from 22 pairs of populations (including bats, shrews, skinks, geckos, snakes, and frogs)

[1] J. R. Oaks et al. (2013). *Evolution* 67: 991–1010. [2] W. Huang et al. (2011). *BMC Bioinformatics* 12: 1.

Previous tests of "species-pump"

- Oaks et al. (2013)[1] collected mitochondrial DNA sequences from 22 pairs of populations (including bats, shrews, skinks, geckos, snakes, and frogs)
  - Analyzed these data with ABC method `msBayes`[2]

[1] J. R. Oaks et al. (2013). *Evolution* 67: 991–1010. [2] W. Huang et al. (2011). *BMC Bioinformatics* 12: 1.

Previous tests of "species-pump"

- Oaks et al. (2013)[1] collected mitochondrial DNA sequences from 22 pairs of populations (including bats, shrews, skinks, geckos, snakes, and frogs)
  - Analyzed these data with ABC method `msBayes`[2]
  - Found strong support for shared divergences across taxa (results below for 9 pairs from islands of Negros and Panay)

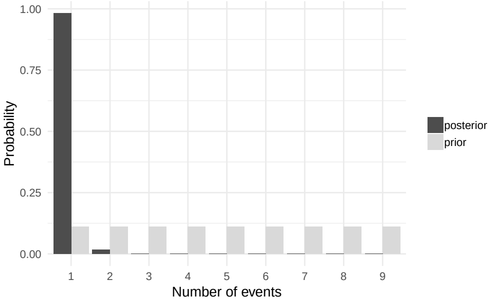

[1] J. R. Oaks et al. (2013). *Evolution* 67: 991–1010. [2] W. Huang et al. (2011). *BMC Bioinformatics* 12: 1.

Previous tests of "species-pump"

- Oaks et al. (2013)[1] collected mitochondrial DNA sequences from 22 pairs of populations (including bats, shrews, skinks, geckos, snakes, and frogs)
  - Analyzed these data with ABC method `msBayes`[2]
  - Found strong support for shared divergences across taxa (results below for 9 pairs from islands of Negros and Panay)
  - But, method was very sensitive to prior assumptions and often biased toward estimating co-divergences

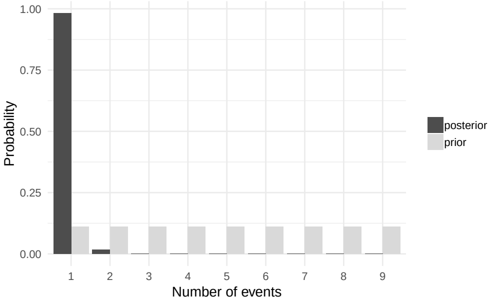

[1] J. R. Oaks et al. (2013). *Evolution* 67: 991–1010. [2] W. Huang et al. (2011). *BMC Bioinformatics* 12: 1.

Previous tests of "species-pump"

- Oaks (2014)[1] reanalyzed the data from Oaks et al. (2013)[2] with a modified ABC method `dpp-msbayes`

[1] J. R. Oaks (2014). *BMC Evolutionary Biology* 14: 150. [2] J. R. Oaks et al. (2013). *Evolution* 67: 991–1010.

Previous tests of “species-pump”

- Oaks (2014)[1] reanalyzed the data from Oaks et al. (2013)[2] with a modified ABC method `dpp-msbayes`
  - New method was less biased, but little information in summary statistics to inform divergence times

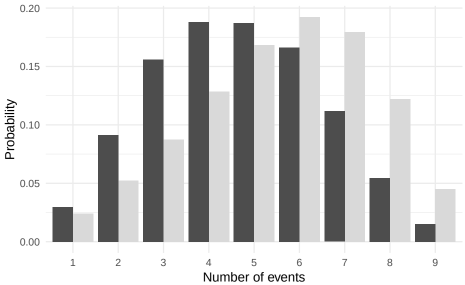

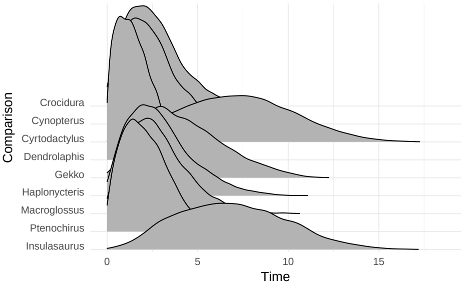

[1] J. R. Oaks (2014). *BMC Evolutionary Biology* 14: 150. [2] J. R. Oaks et al. (2013). *Evolution* 67: 991–1010.

Previous tests of “species-pump”

- Oaks (2014)[1] reanalyzed the data from Oaks et al. (2013)[2] with a modified ABC method `dpp-msbayes`
  - New method was less biased, but little information in summary statistics to inform divergence times

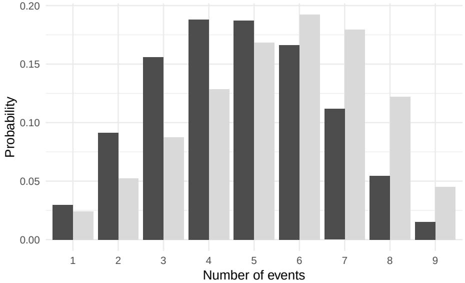

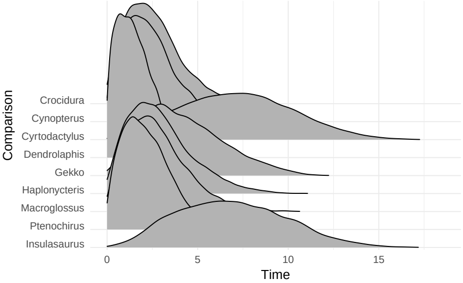

What now? We need **more data** and/or an **improved method** that better utilizes the information in those data.

[1] J. R. Oaks (2014). *BMC Evolutionary Biology* 14: 150. [2] J. R. Oaks et al. (2013). *Evolution* 67: 991–1010.

Previous tests of "species-pump"

- Oaks (2014)[1] reanalyzed the data from Oaks et al. (2013)[2] with a modified ABC method `dpp-msbayes`
  - New method was less biased, but little information in summary statistics to inform divergence times

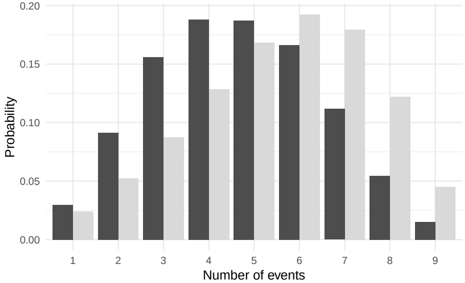

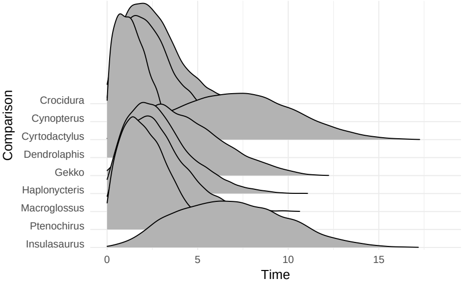

What now? We need **more data** and/or an **improved method** that better utilizes the information in those data. **Our goal is to do both.**

[1] J. R. Oaks (2014). *BMC Evolutionary Biology* 14: 150. [2] J. R. Oaks et al. (2013). *Evolution* 67: 991–1010.

Cyrtodactylus (Gekkonidae)

- 265+ species across Asia
- 10+ species across Philippines
- Nocturnal, scansorial lizards that eat terrestrial invertebrates
- Specialized bent toes for climbing

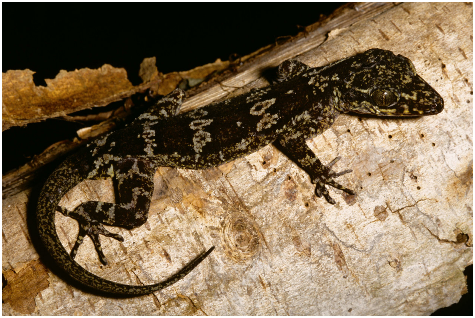

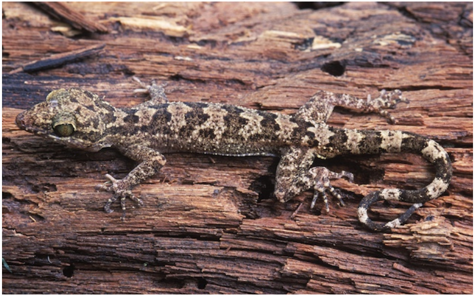

Gekko (Gekkonidae)

- 60+ species across Southeast Asia
- 14+ species across Philippines
- Nocturnal, scansorial lizards that eat terrestrial invertebrates
- Subdigital lamellae for climbing

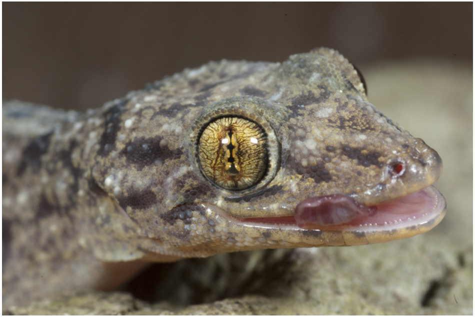

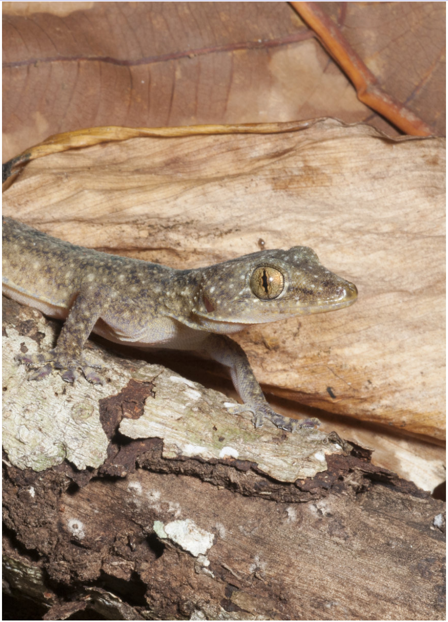

Methods

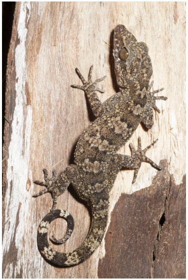

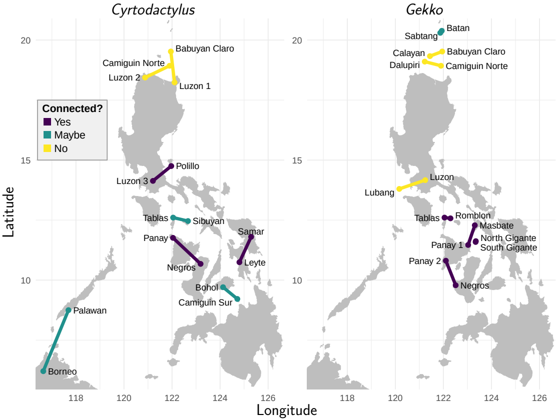

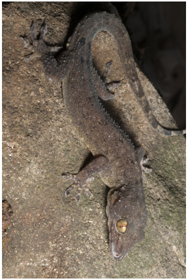

- Sampled individuals from 8 pairs of populations for both *Cyrtodactylus* and *Gekko*
  - Sampled 2–5 individuals per population
- Collected genome-wide DNA sequence data from each individual
  - Restriction-site-associated DNA sequencing (RADseq)

Analyzed RADseq data with full-likelihood Bayesian comparative phylogeographic method:

Ecoevolity: **E**stimating **evo**lutionary **coevality**

Analyzed RADseq data with full-likelihood Bayesian comparative phylogeographic method:

Ecoevolity: **E**stimating **evo**lutionary **coevality**

- Used simulations to assess how well `ecoevolity` works given the gekkonid RADseq data sets

Results: *Cyrtodactylus* (Figure 2)

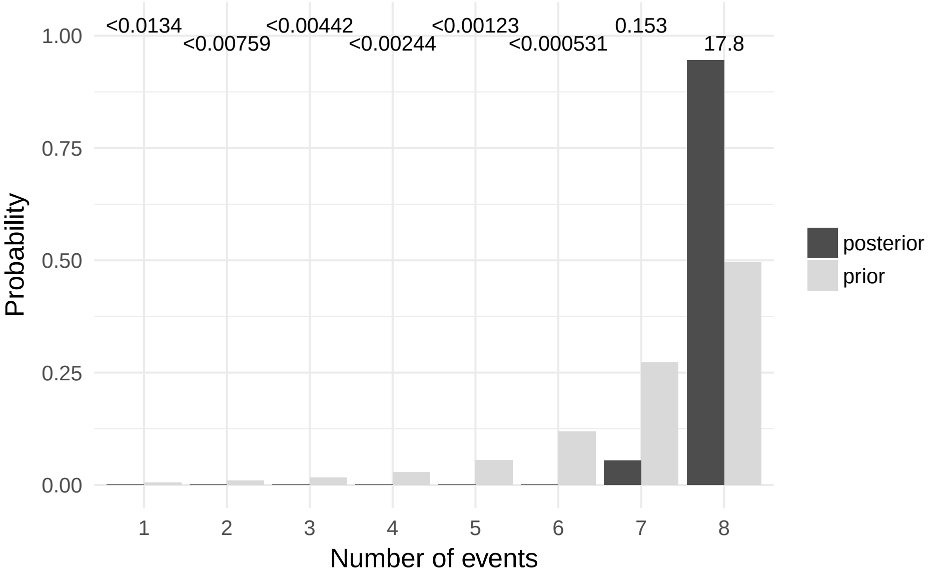

Results: *Cyrtodactylus* (Figure 3)

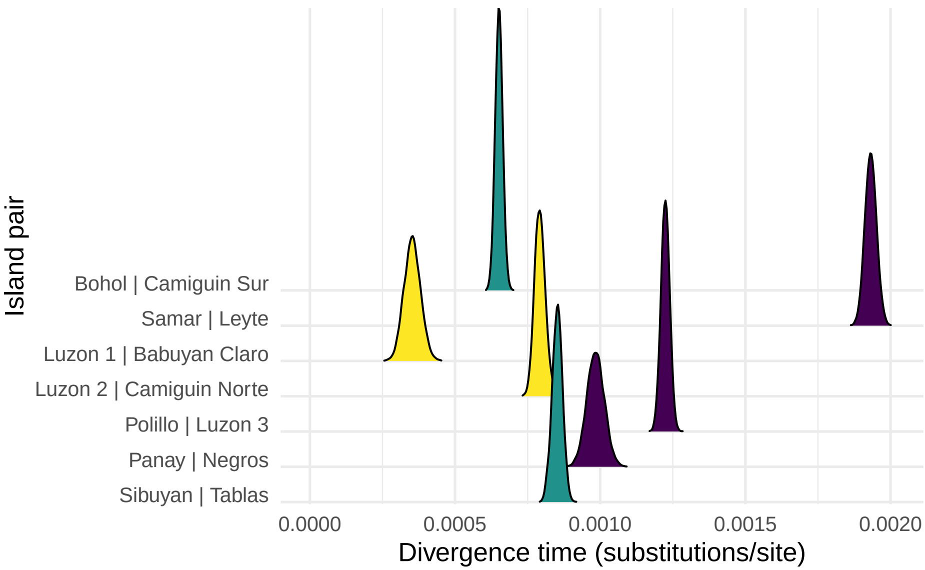

Results: *Gekko* (Figure 4)

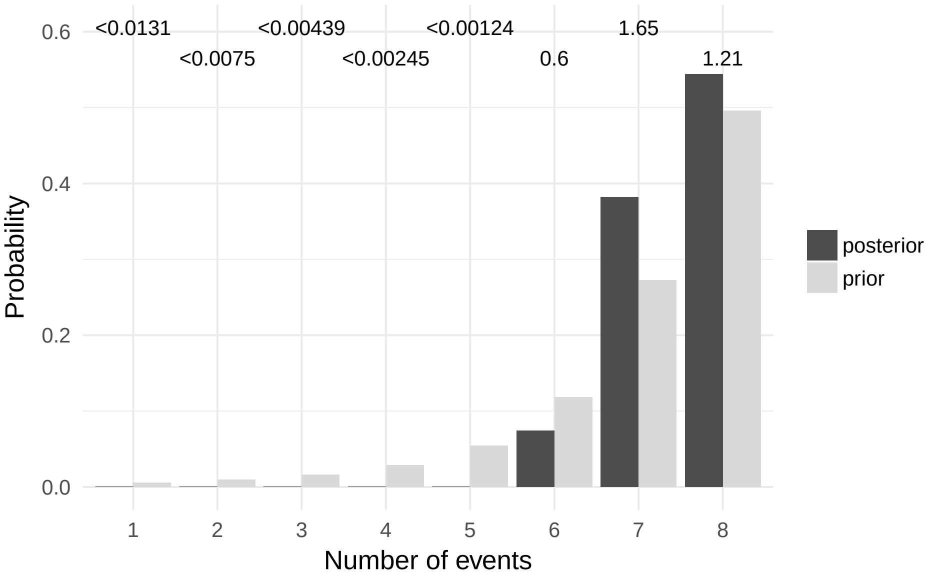

Results: *Gekko* (Figure 5)

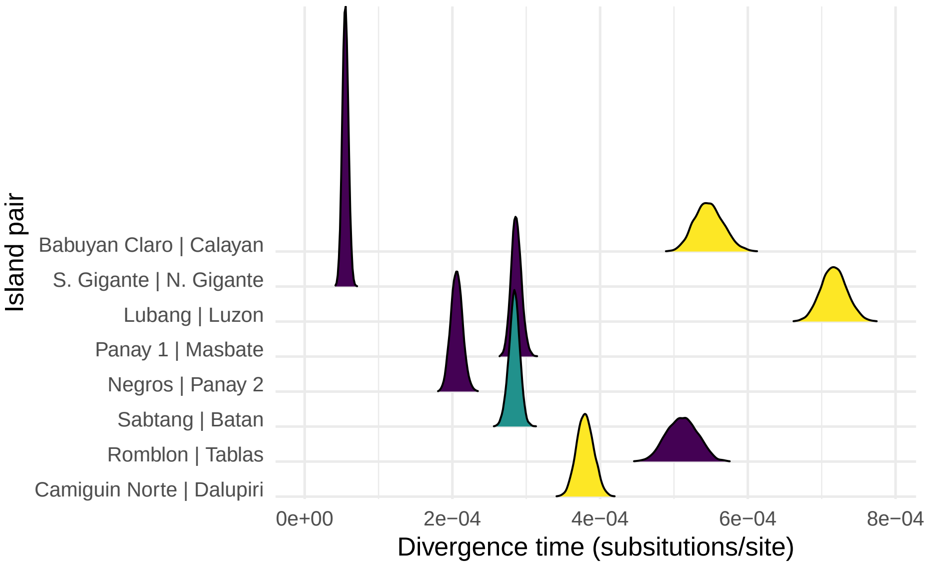

Key findings

- Strong support that all 8 pairs of *Cyrtodactylus* populations diverged independently

Key findings

- Strong support that all 8 pairs of *Cyrtodactylus* populations diverged independently
- Weak support that all 8 pairs of *Gekko* populations diverged independently

Key findings

- Strong support that all 8 pairs of *Cyrtodactylus* populations diverged independently
- Weak support that all 8 pairs of *Gekko* populations diverged independently
- Simulation results suggest `ecoevolity` can accurately estimate the timing and number of divergences given the gekkonid RADseq data

Caveats

- Too few island pairs to rule out climate-driven vicariant speciation
- Differences in divergence times could be due to variation in fragmentation times among island pairs
- Differences in divergence could also be due to variation in mutation rates

Caveats

- Too few island pairs to rule out climate-driven vicariant speciation
- Differences in divergence times could be due to variation in fragmentation times among island pairs
- Differences in divergence could also be due to variation in mutation rates

- Seems safe to conclude that the "species-pump" is not the rule for gekkonids, but maybe the exception

Take home points

- Support against the “species-pump” hypothesis

Take home points

- Support against the "species-pump" hypothesis
- Results suggest repeated cycles of climate-driven island fragmentation were not an important mechanism of speciation for gekkonid lizards in the Philippines

Take home points

- Support against the "species-pump" hypothesis
- Results suggest repeated cycles of climate-driven island fragmentation were not an important mechanism of speciation for gekkonid lizards in the Philippines
- Rare over-water dispersal via rafting on vegetation is likely an important mechanism responsible for the distribution of gekkonid lizards in the Philippines

Everything is on GitHub…

Software:

- Ecoevolity: https://github.com/phyletica/ecoevolity

Open-Science Notebook:

- Gecko RADseq: https://github.com/phyletica/gekgo

Acknowledgments

Ideas and feedback:

- Phyletica Lab (the Phyleticians)
- Constructive feedback from Editor-in-Chief Mohamed Noor, Associate Editor David Weisrock, and four anonymous reviewers greatly improved this work

Lab work:

- Patrick Monnahan and John Kelly for their help with the MSG libraries

Computation:

- Alabama Supercomputer Authority
- Auburn University Hopper Cluster

Funding:

Photo credits:

- Rafe Brown and Cam Siler
- PhyloPic!

Questions?

joaks@auburn.edu

phyletica.org

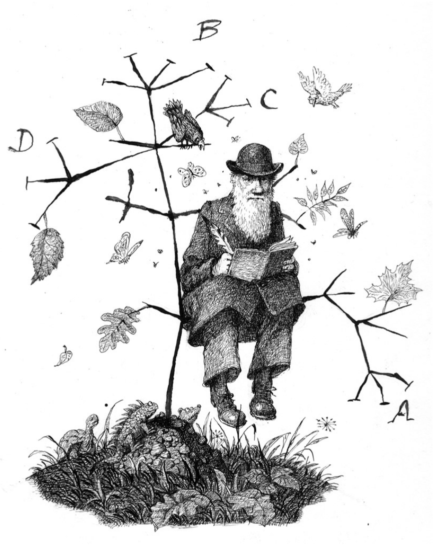

© 2007 Boris Kulikov boris-kulikov.blogspot.com

Figure 6

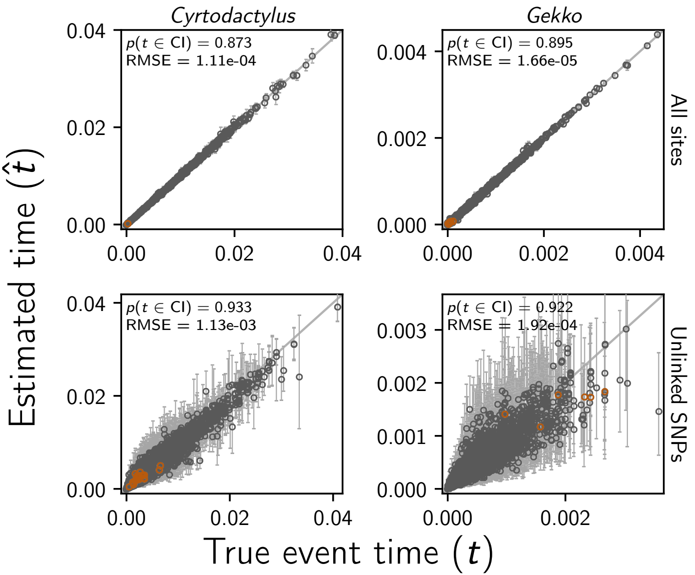

Cyrtodactylus
Gekko
$p(t \in \text{CI}) = 0.873$
RMSE = 1.11e-04
$p(t \in \text{CI}) = 0.895$
RMSE = 1.66e-05
$p(t \in \text{CI}) = 0.933$
RMSE = 1.13e-03
$p(t \in \text{CI}) = 0.922$
RMSE = 1.92e-04
All sites
Unlinked SNPs
Estimated time ($\hat{t}$)
True event time (t)
0.00
0.02
0.04
0.000
0.001
0.002
0.003
0.004

Figure 7

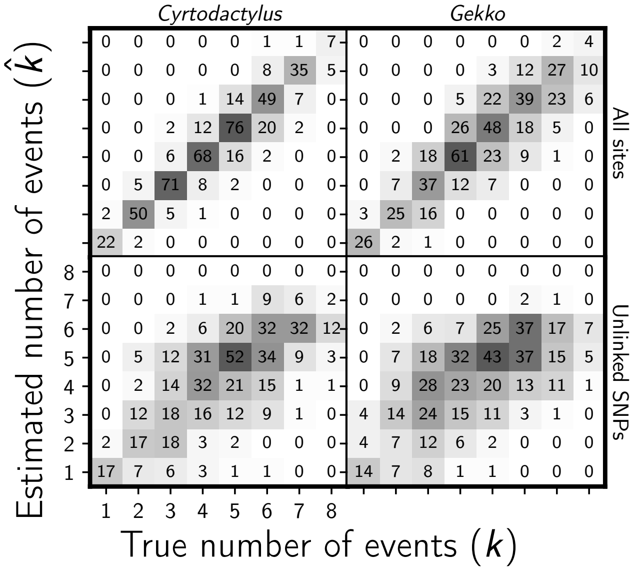
Cyrtodactylus
Gekko
Estimated number of events ($\hat{k}$)
True number of events (k)
All sites
Unlinked SNPs
0 0 0 0 0 1 1 7 0 0 0 0 0 0 2 4
0 0 0 0 0 8 35 5 0 0 0 0 3 12 27 10
0 0 0 1 14 49 7 0 0 0 0 5 22 39 23 6
0 0 2 12 76 20 2 0 0 0 0 26 48 18 5 0
0 0 6 68 16 2 0 0 0 2 18 61 23 9 1 0
0 5 71 8 2 0 0 0 0 7 37 12 7 0 0 0
2 50 5 1 0 0 0 0 3 25 16 0 0 0 0 0
22 2 0 0 0 0 0 0 26 2 1 0 0 0 0 0
8 0 0 0 0 0 0 0 0 0 0 0 0 0 0 0 0
7 0 0 0 1 1 9 6 2 0 0 0 0 0 2 1 0
6 0 0 2 6 20 32 32 12 0 2 6 7 25 37 17 7
5 0 5 12 31 52 34 9 3 0 7 18 32 43 37 15 5
4 0 2 14 32 21 15 1 1 0 9 28 23 20 13 11 1
3 0 12 18 16 12 9 1 0 4 14 24 15 11 3 1 0
2 2 17 18 3 2 0 0 0 4 7 12 6 2 0 0 0
1 17 7 6 3 1 1 0 0 14 7 8 1 1 0 0 0
1 2 3 4 5 6 7 8

Figure S1

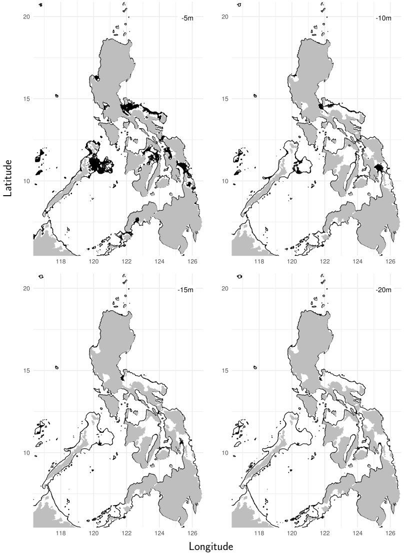

Figure S2

Click here for a sea-level animation of SE Asia

Figure S3

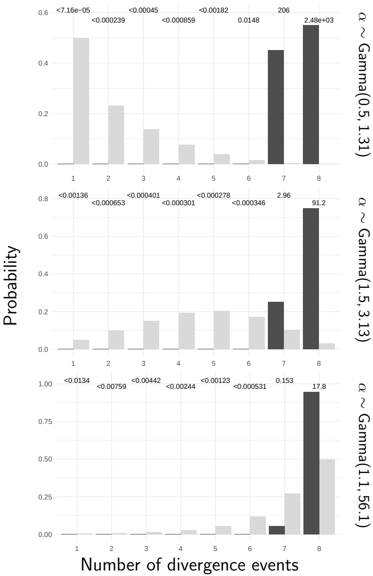

Probability
Number of divergence events
α ~ Gamma(0.5, 1.31)
α ~ Gamma(1.5, 3.13)
α ~ Gamma(1.1, 56.1)
<7.16e−05
<0.000239
<0.00045
<0.000859
<0.00182
0.0148
206
2.48e+03
<0.00136
<0.000653
<0.000401
<0.000301
<0.000278
<0.000346
2.96
91.2
<0.0134
<0.00759
<0.00442
<0.00244
<0.00123
<0.000531
0.153
17.8

Figure S4

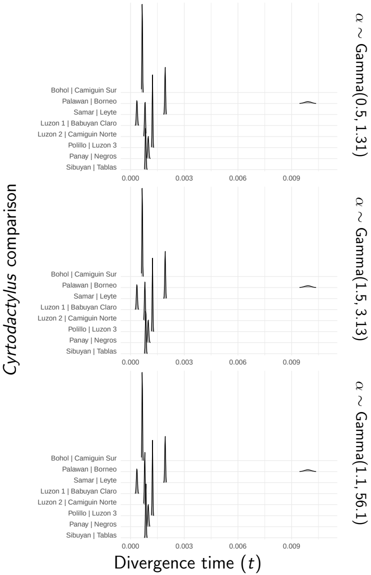
Cyrtodactylus comparison
Bohol | Camiguin Sur
Palawan | Borneo
Samar | Leyte
Luzon 1 | Babuyan Claro
Luzon 2 | Camiguin Norte
Polillo | Luzon 3
Panay | Negros
Sibuyan | Tablas
0.000
0.003
0.006
0.009
α ~ Gamma(0.5, 1.31)
α ~ Gamma(1.5, 3.13)
α ~ Gamma(1.1, 56.1)
Divergence time (t)

Figure S5

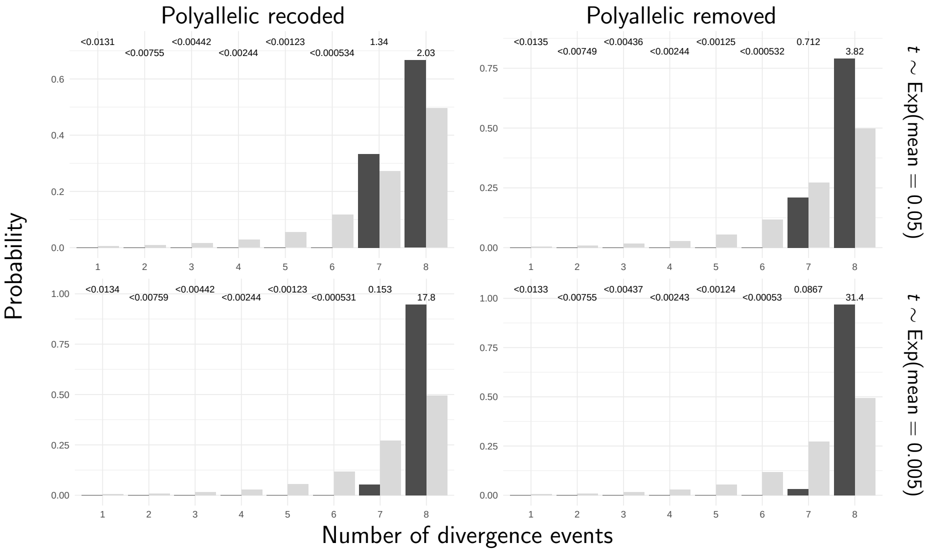

Polyallelic recoded
Polyallelic removed
<0.0131
<0.00755
<0.00442
<0.00244
<0.00123
<0.000534
1.34
2.03
<0.0135
<0.00749
<0.00436
<0.00244
<0.00125
<0.000532
0.712
3.82
<0.0134
<0.00759
<0.00442
<0.00244
<0.00123
<0.000531
0.153
17.8
<0.0133
<0.00755
<0.00437
<0.00243
<0.00124
<0.00053
0.0867
31.4
Probability
Number of divergence events
$t \sim \text{Exp}(\text{mean} = 0.05)$
$t \sim \text{Exp}(\text{mean} = 0.005)$

Figure S6

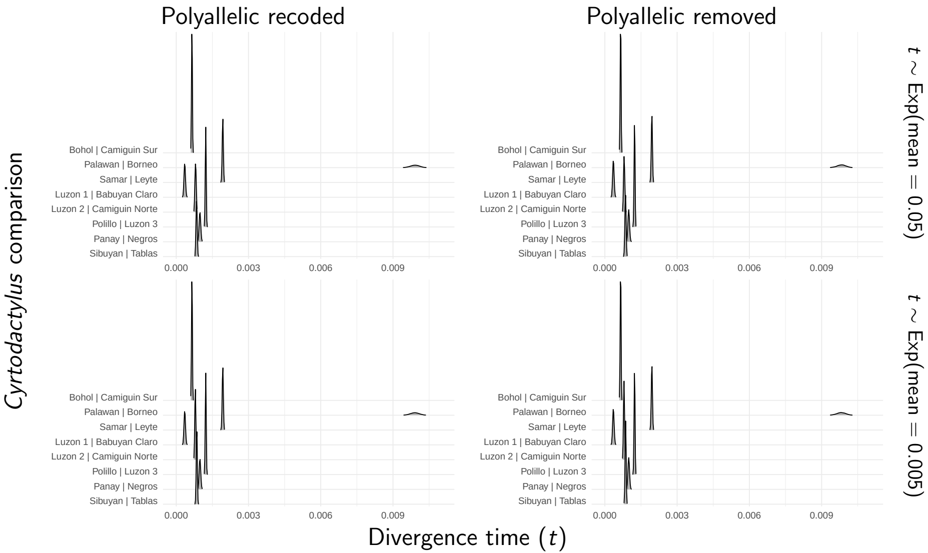
Polyallelic recoded
Polyallelic removed
Bohol | Camiguin Sur
Palawan | Borneo
Samar | Leyte
Luzon 1 | Babuyan Claro
Luzon 2 | Camiguin Norte
Polillo | Luzon 3
Panay | Negros
Sibuyan | Tablas
0.000
0.003
0.006
0.009
Cyrtodactylus comparison
Divergence time (t)
t ~ Exp(mean = 0.05)
t ~ Exp(mean = 0.005)

Figure S7

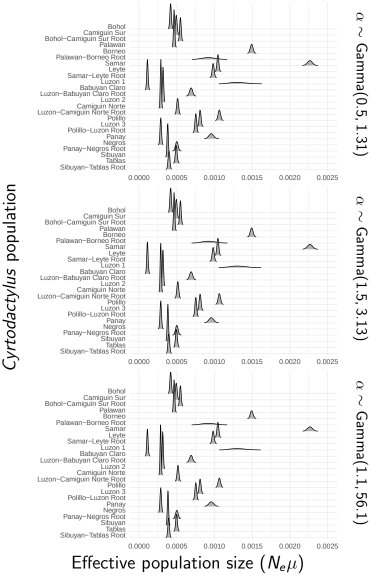
Cyrtodactylus population
Bohol
Camiguin Sur
Bohol−Camiguin Sur Root
Palawan
Borneo
Palawan−Borneo Root
Samar
Leyte
Samar−Leyte Root
Luzon 1
Babuyan Claro
Luzon−Babuyan Claro Root
Luzon 2
Camiguin Norte
Luzon−Camiguin Norte Root
Polillo
Luzon 3
Polillo−Luzon Root
Panay
Negros
Panay−Negros Root
Sibuyan
Tablas
Sibuyan−Tablas Root
0.0000
0.0005
0.0010
0.0015
0.0020
0.0025
$\alpha \sim$ Gamma(0.5, 1.31)
$\alpha \sim$ Gamma(1.5, 3.13)
$\alpha \sim$ Gamma(1.1, 56.1)
Effective population size ($N_e\mu$)

Figure S8

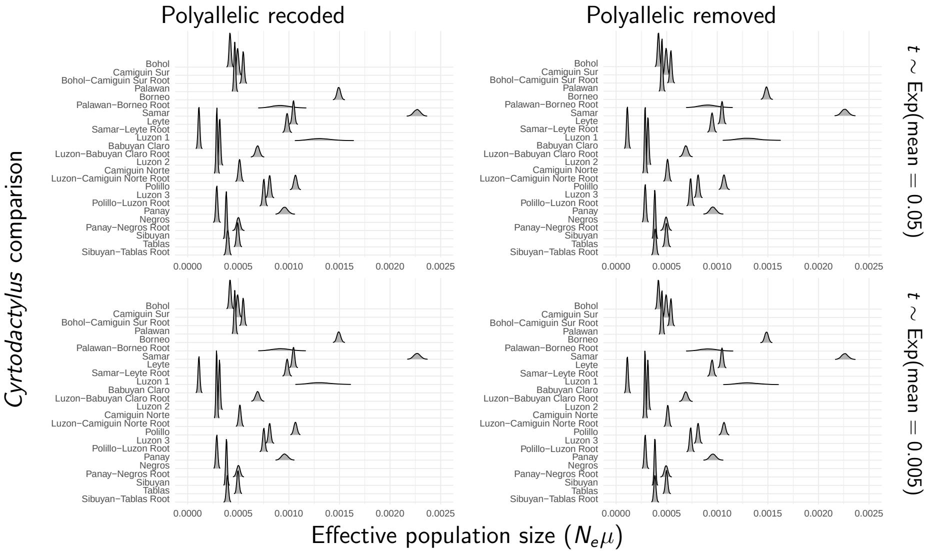

Polyallelic recoded
Polyallelic removed
Bohol
Camiguin Sur
Bohol−Camiguin Sur Root
Palawan
Borneo
Palawan−Borneo Root
Samar
Leyte
Samar−Leyte Root
Luzon 1
Babuyan Claro
Luzon−Babuyan Claro Root
Luzon 2
Camiguin Norte
Luzon−Camiguin Norte Root
Polillo
Luzon 3
Polillo−Luzon Root
Panay
Negros
Panay−Negros Root
Sibuyan
Tablas
Sibuyan−Tablas Root
0.0000
0.0005
0.0010
0.0015
0.0020
0.0025
Cyrtodactylus comparison
$t \sim$ Exp(mean = 0.05)
$t \sim$ Exp(mean = 0.005)
Effective population size ($N_e\mu$)

Figure S9

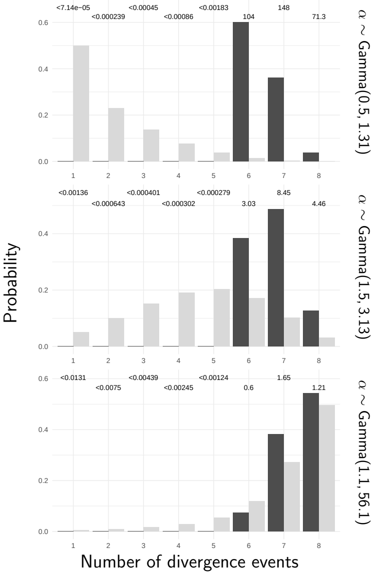

<7.14e−05
<0.000239
<0.00045
<0.00086
<0.00183
104
148
71.3
<0.00136
<0.000643
<0.000401
<0.000302
<0.000279
3.03
8.45
4.46
<0.0131
<0.0075
<0.00439
<0.00245
<0.00124
0.6
1.65
1.21
Probability
Number of divergence events
α ∼ Gamma(0.5, 1.31)
α ∼ Gamma(1.5, 3.13)
α ∼ Gamma(1.1, 56.1)

Figure S10

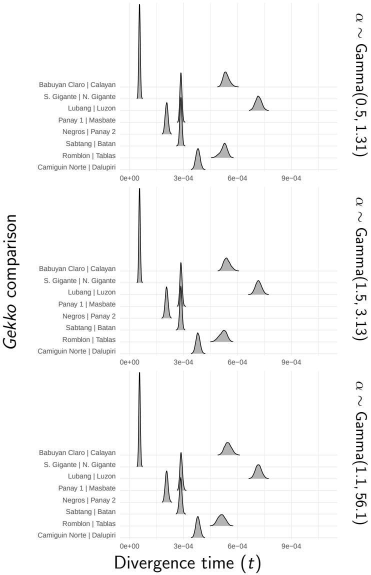
Gekko comparison
Babuyan Claro | Calayan
S. Gigante | N. Gigante
Lubang | Luzon
Panay 1 | Masbate
Negros | Panay 2
Sabtang | Batan
Romblon | Tablas
Camiguin Norte | Dalupiri
0e+00
3e−04
6e−04
9e−04
α ~ Gamma(0.5, 1.31)
α ~ Gamma(1.5, 3.13)
α ~ Gamma(1.1, 56.1)
Divergence time (t)

Figure S11

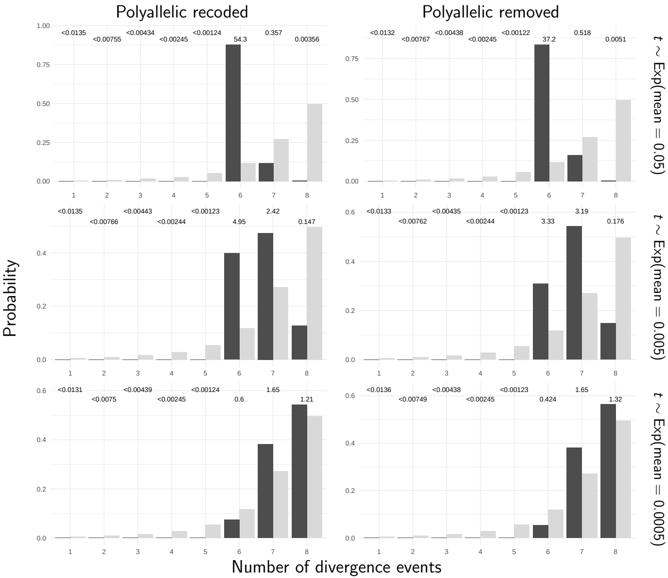
Polyallelic recoded
Polyallelic removed
Probability
Number of divergence events
t ∼ Exp(mean = 0.05)
t ∼ Exp(mean = 0.005)
t ∼ Exp(mean = 0.0005)
<0.0135 <0.00755 <0.00434 <0.00245 <0.00124 54.3 0.357 0.00356
<0.0132 <0.00767 <0.00438 <0.00245 <0.00122 37.2 0.518 0.0051
<0.0135 <0.00766 <0.00443 <0.00244 <0.00123 4.95 2.42 0.147
<0.0133 <0.00762 <0.00435 <0.00244 <0.00123 3.33 3.19 0.176
<0.0131 <0.0075 <0.00439 <0.00245 <0.00124 0.6 1.65 1.21
<0.0136 <0.00749 <0.00438 <0.00245 <0.00123 0.424 1.65 1.32
1 2 3 4 5 6 7 8

Figure S12

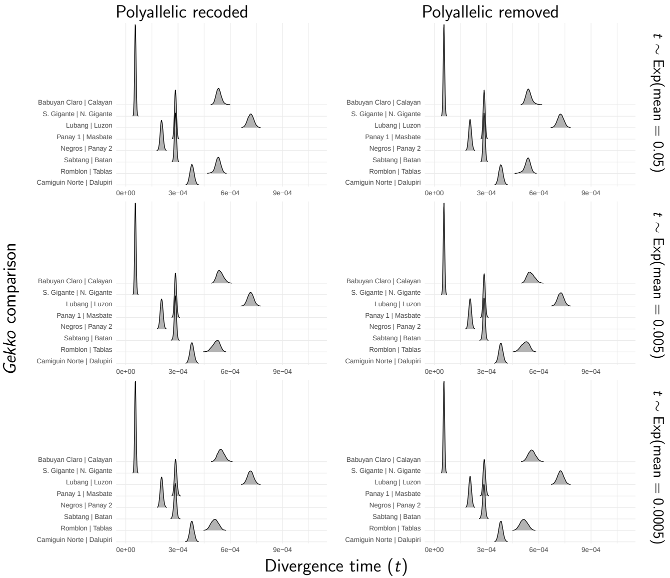

Polyallelic recoded
Polyallelic removed
Gekko comparison
Babuyan Claro | Calayan
S. Gigante | N. Gigante
Lubang | Luzon
Panay 1 | Masbate
Negros | Panay 2
Sabtang | Batan
Romblon | Tablas
Camiguin Norte | Dalupiri
0e+00
3e−04
6e−04
9e−04
t ∼ Exp(mean = 0.05)
t ∼ Exp(mean = 0.005)
t ∼ Exp(mean = 0.0005)
Divergence time (t)

Figure S13

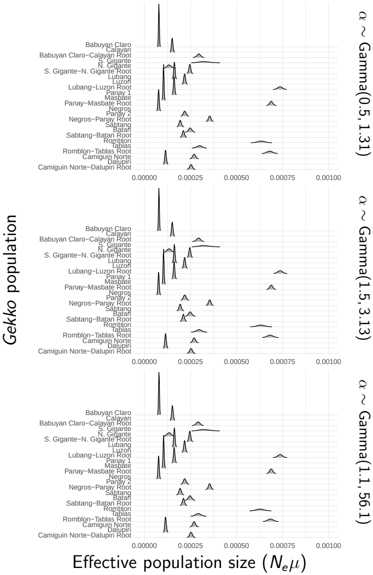
Gekko population
Babuyan Claro
Calayan
Babuyan Claro−Calayan Root
S. Gigante
N. Gigante
S. Gigante−N. Gigante Root
Lubang
Luzon
Lubang−Luzon Root
Panay 1
Masbate
Panay−Masbate Root
Negros
Panay 2
Negros−Panay Root
Sabtang
Batan
Sabtang−Batan Root
Romblon
Tablas
Romblon−Tablas Root
Camiguin Norte
Dalupiri
Camiguin Norte−Dalupiri Root
0.00000
0.00025
0.00050
0.00075
0.00100
$\alpha \sim$ Gamma(0.5, 1.31)
$\alpha \sim$ Gamma(1.5, 3.13)
$\alpha \sim$ Gamma(1.1, 56.1)
Effective population size ($N_e\mu$)

Figure S14

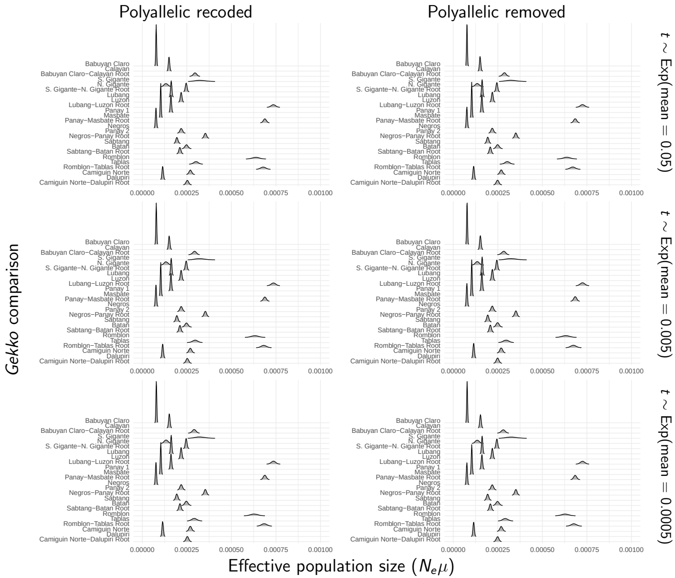
Polyallelic recoded
Polyallelic removed
Babuyan Claro
Calayan
Babuyan Claro−Calayan Root
S. Gigante
N. Gigante
S. Gigante−N. Gigante Root
Lubang
Luzon
Lubang−Luzon Root
Panay 1
Masbate
Panay−Masbate Root
Negros
Panay 2
Negros−Panay Root
Sabtang
Batan
Sabtang−Batan Root
Romblon
Tablas
Romblon−Tablas Root
Camiguin Norte
Dalupiri
Camiguin Norte−Dalupiri Root
0.00000
0.00025
0.00050
0.00075
0.00100
Gekko comparison
$t \sim \text{Exp}(\text{mean} = 0.05)$
$t \sim \text{Exp}(\text{mean} = 0.005)$
$t \sim \text{Exp}(\text{mean} = 0.0005)$
Effective population size ($N_e\mu$)

Figure S15

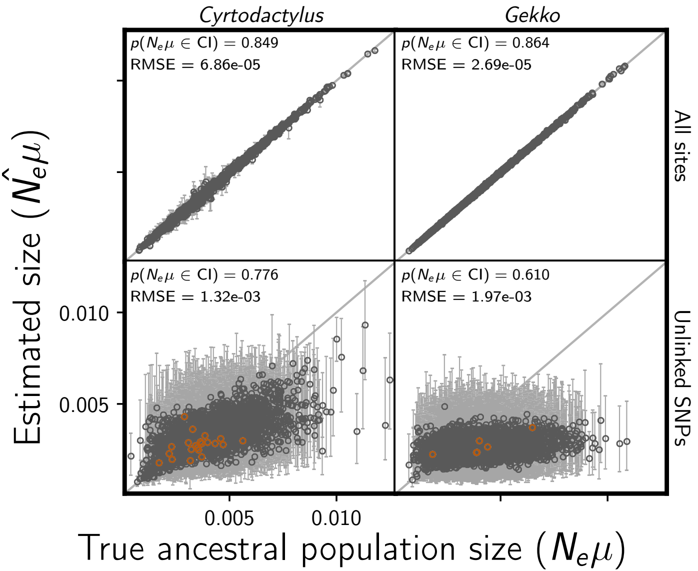

Cyrtodactylus
Gekko
$p(N_e\mu \in \text{CI}) = 0.849$
RMSE = 6.86e-05
$p(N_e\mu \in \text{CI}) = 0.864$
RMSE = 2.69e-05
$p(N_e\mu \in \text{CI}) = 0.776$
RMSE = 1.32e-03
$p(N_e\mu \in \text{CI}) = 0.610$
RMSE = 1.97e-03
All sites
Unlinked SNPs
0.010
0.005
0.005
0.010
Estimated size ($\hat{N_e\mu}$)
True ancestral population size ($N_e\mu$)

Figure S16

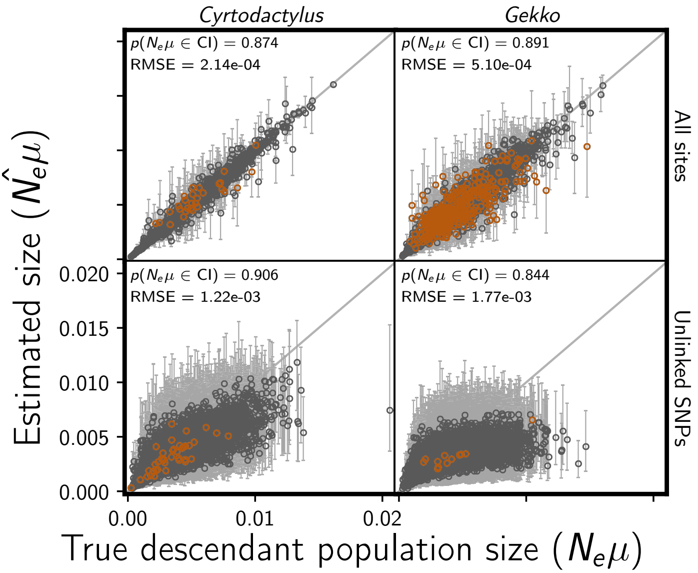
Cyrtodactylus
Gekko
$p(N_e\mu \in \text{CI}) = 0.874$
RMSE = 2.14e-04
$p(N_e\mu \in \text{CI}) = 0.891$
RMSE = 5.10e-04
$p(N_e\mu \in \text{CI}) = 0.906$
RMSE = 1.22e-03
$p(N_e\mu \in \text{CI}) = 0.844$
RMSE = 1.77e-03
All sites
Unlinked SNPs
Estimated size ($\hat{N_e\mu}$)
0.020
0.015
0.010
0.005
0.000
0.00
0.01
0.02
True descendant population size ($N_e\mu$)

Figure S17

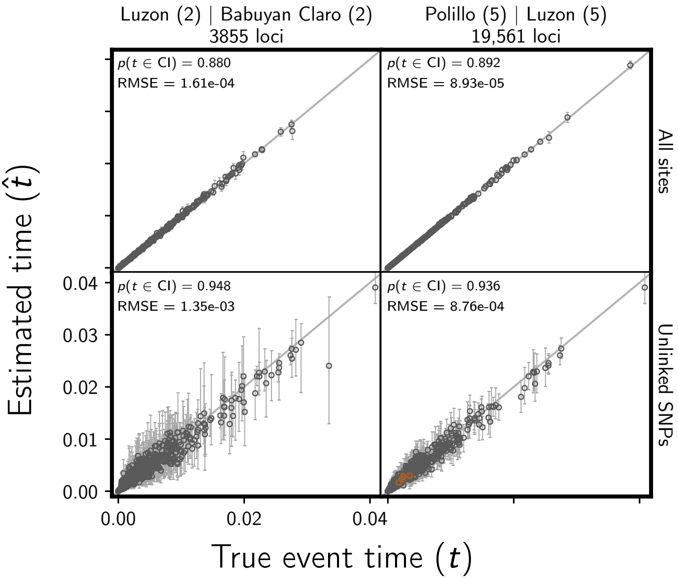

Luzon (2) | Babuyan Claro (2)
3855 loci
Polillo (5) | Luzon (5)
19,561 loci
$p(t \in \mathrm{CI}) = 0.880$
RMSE = 1.61e-04
$p(t \in \mathrm{CI}) = 0.892$
RMSE = 8.93e-05
$p(t \in \mathrm{CI}) = 0.948$
RMSE = 1.35e-03
$p(t \in \mathrm{CI}) = 0.936$
RMSE = 8.76e-04
All sites
Unlinked SNPs
Estimated time ($\hat{t}$)
True event time (t)
0.00
0.01
0.02
0.03
0.04
0.00
0.02
0.04
